# Can living donor liver transplantation provide similar outcomes to deceased-donor liver transplantation for hepatocellular carcinoma? A systematic review and meta-analysis

**DOI:** 10.1007/s12072-022-10435-3

**Published:** 2022-12-23

**Authors:** Beshoy Effat Elkomos, Mostafa Abdo, Remon Mamdouh, Amr Abdelaal

**Affiliations:** General Surgery Department, Ain Shams University Hospital, Cairo, Egypt

**Keywords:** Liver transplantation, Living donor, Living donor liver transplantation, Deceased donor, Deceased donor liver transplantation, Hepatocellular carcinoma, Cancer liver, Liver tumor, LT, Hepatobiliary surgery

## Abstract

**Background and Aim:**

A potential solution to the deceased organ shortage is to include live organ donations and to identify patients with lower rates of HCC recurrence to fairly allocate liver grafts. Our aims were to detect the long-term outcomes of LDLT versus DDLT for HCC and predictors of recurrence after transplantation.

**Methods:**

PubMed, Scopus, Web of Science, Cochrane library were searched for eligible studies from inception to July 2021 and a systematic review and meta-analysis were done.

**Results:**

35 studies with a total of 7822 patients were included. The 1-, 3-, 4 year-OS showed trivial improvement for LDLT recipients. However, the two modalities had similar 5-, 6- and 10-year OS. A significant improvement in the ITT-OS was observed for LDLT recipients. Regarding the DFS and recurrence after transplantation, no significant difference was observed between LDLT and DDLT. In addition to that, the pooled hazard ratio of the included studies showed that Milan criteria, level of AFP, presence of vascular invasion, tumor differentiation were significant predictors of recurrence.

**Conclusion:**

The cancer biology (not the graft type) is the most important determinant of recurrence and survival after LT. However, LDLT provided much better survival benefits to HCC patients especially in regions that suffer from low deceased organ availability.

**Supplementary Information:**

The online version contains supplementary material available at 10.1007/s12072-022-10435-3.

## Introduction

Liver cancer remains a global health problem and its incidence is rising worldwide [1, 2]. It is estimated that, by 2025, > 1 million people will be diagnosed with liver cancer annually [3]. Hepatocellular carcinoma (HCC) is the most common form of primary liver cancer and accounts for ~ 90% of cases [4].

Therapeutic treatment options are available for patients with the local disease and include ablation, resection, and liver transplantation (LT) [5]. LT is a recognized treatment choice for patients with cirrhosis of the liver and HCC [6].

The greatest obstacle in liver transplant is the shortage of donors which has contributed to a remarkable increase in the waiting lists. Therefore, there is an increase in the time from the decision of transplantation to the LT itself. During this period, the HCC may progress and drop out from the waiting list [7–9].

Several strategies have been evaluated to reduce this risk: increasing the pool of donors by including live donors, treatment of HCC upon enlistment, and priority policies by identifying patients with lower rates of HCC recurrence and higher rates of survival to fairly allocate liver grafts. However, the long-term outcomes of LDLT versus DDLT for HCC are still controversial. Several studies demonstrate that LDLT was associated with better intention to treat overall survival (ITT-OS) when compared to DDLT [10, 11]. While some studies illustrated that HCC patients undergoing LDLT would result in worse DFS and recurrence rate [12, 13], other studies reported equal recurrence rate for the two modalities [14]. Moreover, some studies showed equal overall survival and DFS between the two modalities [10]. In addition to that, there are many predictors of recurrence other than the type of the graft such as level of AFP, vascular invasion and tumor grade that could be used to fairly allocate graft to those with lower incidence of recurrence [15–17].

## Patients and methods

### Search strategy

The protocol for this meta-analysis was registered to PROSPERO (CRD42021281670). The search was directed through PubMed, Scopus, Web of science, and the Cochrane Library for information from May 1963 to July 1, 2021 with a combination of the following terms: liver donor liver transplant, hepatocellular carcinoma, LDLT and HCC. More searches by Google Scholar have been used to supplement the search with the sites mentioned above. All studies were reviewed and evaluated by two authors (Elkomos, B. E.& Abdelaal, A.) according to the eligibility process. Abstract-based eligibility studies were obtained, and the manuscripts were fully reviewed.

### Inclusion and exclusion criteria

The eligible studies included the following: (1) randomized controlled trials and prospective or retrospective cohort studies; (2) target population were patients diagnosed with HCC; (3) studies designating a comparison of LDLT and DDLT as a primary aim; and (4) the primary outcomes were overall survival (OS), intention-to-treat overall survival (ITT-OS), disease-free survival (DFS) or recurrence of HCC for both LDLT and DDLT patients. Exclusion criteria: (1) reviews, case reports and case series; (2) studies designed to analyses information from the United Network for Organ Sharing database or the Scientific Registry of Transplant Recipients database; (3) studies missing a comparison group (DDLT recipients).

### Outcomes of interest

We assessed 4 primary outcomes of LDLT and DDLT for HCC patients in this meta-analysis, including patient long-term overall survival from the time of transplant (1-, 2-, 3-, 4-,5-, 6- and 10-year OS), patient long-term overall survival from the time of listing to transplantation (1-, 2-, 3-, 4-,5-, 6- and 10-year ITT-OS), disease-free survival (1-, 2-, 3-, 4-,5-, 6- and 10-year DFS) and recurrence rate. In addition to that, our secondary outcomes were to detect the effect of age of recipient, sex of recipient, level of AFP and tumor biology (presence of vascular invasion and tumor grade) on the survival and recurrence of HCC after transplantation.

### Quality assessment and data extraction

A modification of the Newcastle–Ottawa scale was used to assess the quality of all cohort studies included in this meta-analysis [18]. Only studies with seven or more stares were included (Table 2).

We extracted data on study characteristics (author, year of publication, country of transplant, number of institutes included in the study, the follow-up of the patients), patient characteristics (type of graft, sample size, age, gender ratio, wait-time on the listing to transplantation, number of tumor nodules, size of the largest one, Child score, tumor differentiation, vascular invasion, pre-transplant treatment), study primary outcomes and study secondary outcomes. The data were extracted by 2 investigators (Elkomos, B. E.& Abdelaal, A.) independently.

### Statistical analysis

The meta-analysis was performed according to Cochrane Handbook for Systematic Reviews of Interventions [19], which is recommended by the Cochrane Collaboration. Regarding the primary outcomes (OS, DFS, ITT-OS, recurrence of HCC), the pooled risk ratios (RRs) and their corresponding 95% confidence intervals (CIs) were calculated with fixed effects models. However, if there was moderate or considerable heterogeneity (*I*^2^ > 40), random effects models were used to solve the heterogeneity between studies. Nevertheless, pooled hazard ratio were calculated for secondary outcomes (predictors of recurrence and prognostic facts after transplantation). All calculations for the current meta-analysis were performed with Review Manager 5.4 for Windows (Cochrane Collaboration, Oxford, United Kingdom).

### Assessment of publication bias and heterogeneity

Funnel plots were generated so that we could visually inspect for publication bias. Statistical heterogeneity was assessed with forest plots and the inconsistency statistic (*I*^2^). An *I*^2^ value of 40% or less corresponded to low heterogeneity. Statistical significance was considered at *p* < 0.05.

## Results

### Characteristics and quality assessment of eligible studies

**Fig. 1:**
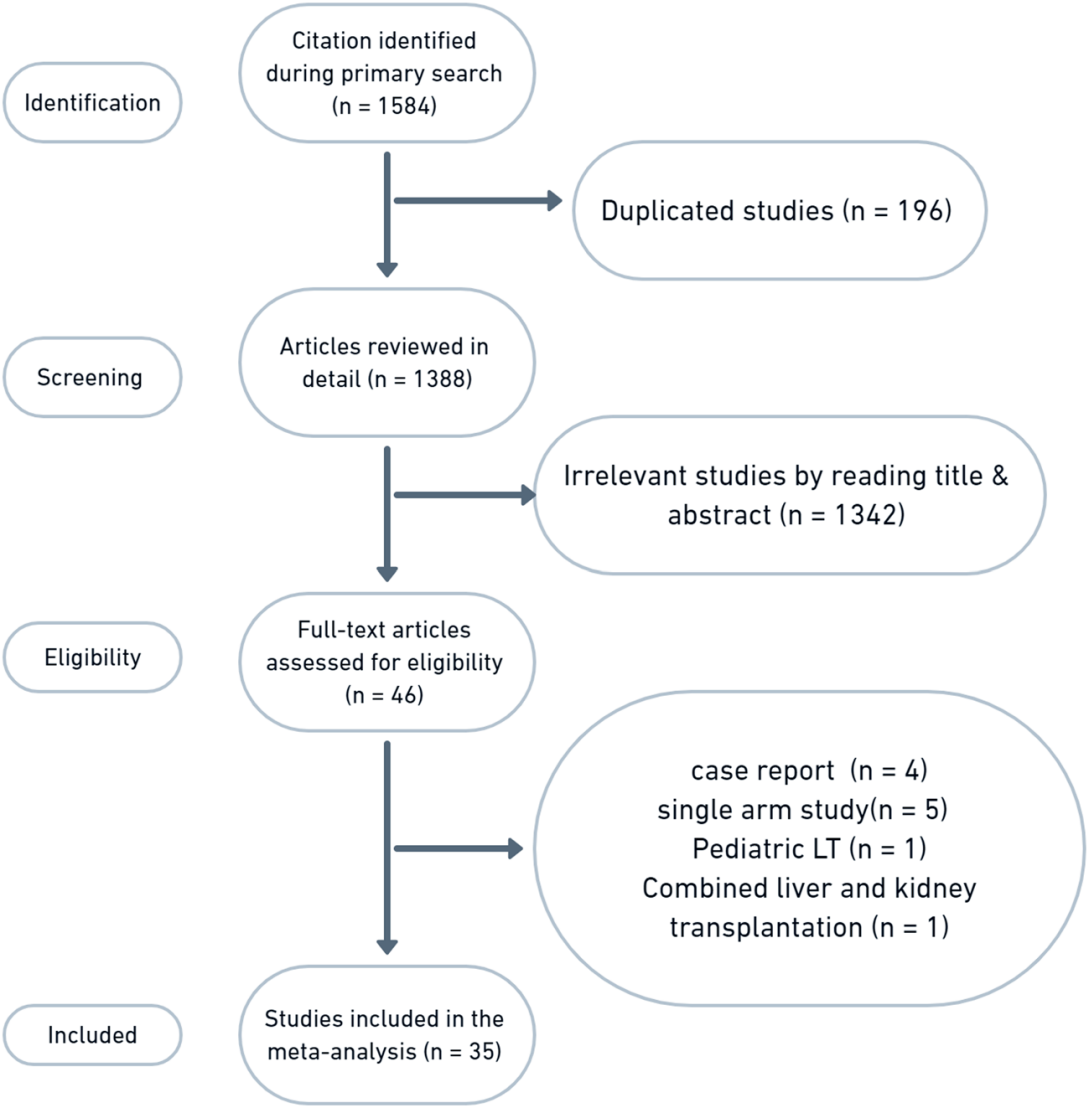
PRISMA flow diagram

As shown in the flow diagram (Fig. 1), 1584 articles were revealed using the following search string: living donor liver transplantation or LDLT and hepatocellular carcinoma or HCC. After careful selection according to our eligibility criteria, 35 controlled clinical trials with 7822 participants were included in the meta-analysis. These trials included 34 retrospective cohort studies and 1 prospective study. However, none of the included studies were randomized studies.

Recipients baseline data [including number, age, sex and waiting time], follow-up time and the tumor-related baseline variables [including percentage of patients beyond the Milan or UCSF criteria, number of tumors, tumor differentiation, the size of largest tumor, vascular invasion, MELD score, Child-Pugh class, and treatment before LT] were comparable between groups in all studies (Table 1). The quality assessment was conducted according to a modification of the Newcastle–Ottawa scale (Table 2). Most of the cohort studies included in this analysis demonstrated sufficient quality with reasonable selection criteria, comparable patient characteristics, and adequate follow-up of the subjects.

**Table 1:** Basic data of the included studies

Studies: author, year, country	Study design	Study period	No. of centers	Arm	Sample size (*n*)	Age (years)	Gender: male/female (*n*)	Waiting time (days)	Follow-up period (years)	Serology: HBV/HCV/both/none (*n*)	Child–Pugh class: A/B/C(*n*)	Within/beyond Milan criteria (*n*)	Within/beyond UCSF criteria (*n*)	No/micro/macro vascular invasion (*n*)	No. of tumor nodules (1/2/3 or more) (*n*)	Size of the largest nodule (cm)	Differentiation: well/moderate/poor (*n*)	Pretreatment: yes/none (*n*)
Gondolesi GE (2004) USA [20]	Retrospective cohort	1998–2002	1	LDLT	36	56.17 ± 7.56^a^	29/7	62	1.25 ± 0.84^a^	9/24/0/3	12/16/8	N/A	N/A	15/15/6	15/8/12	N/A	15/15/6	13/23
		1992–2002		DDLT	165	N/A	N/A	N/A	N/A	N/A	N/A	N/A	N/A	N/A	N/A	N/A	N/A	N/A
Roayaie S (2004) USA [21]	Retrospective cohort	1988–2002	1	LDLT	36	N/A	N/A	N/A	N/A	N/A	N/A	N/A	N/A	N/A	N/A	N/A	N/A	N/A
				DDLT	275	N/A	N/A	N/A	N/A	N/A	N/A	N/A	N/A	N/A	N/A	N/A	N/A	N/A
Hwang S (2005) Korea [22]	Retrospective cohort	1992–2002	4	LDLT	237	50 ± 8^a^	196/41	N/A	3.75 (0.3–8.4)^b^	215/13/8/1	29/70/138	173/64	N/A	N/A	N/A	N/A	N/A	N/A
				DDLT	75	49 ± 7^a^	60/15	N/A	2.2 (0.3–6.7)^b^	68/6/0/1	4/13/55	53/22	N/A	N/A	N/A	N/A	N/A	N/A
Karakayali H (2006) Turkey [23]	Retrospective cohort	2004–2005	1	LDLT	11	31.8 ± 24.9^a^	N/A	N/A	N/A	2/4/0/5	N/A	N/A	N/A	N/A	N/A	N/A	N/A	9/2
				DDLT	6	55 ± 4.7^a^	N/A	N/A	N/A	5/0/0/1	N/A	N/A	N/A	N/A	N/A	N/A	N/A	3/3
Sotiropoulos GC (2007) Germany [24]	Retrospective cohort	1998–2006	1	LDLT	45	55.0 ± 10.1^a^	33/12	N/A	N/A	12/18/2/13	10/24/11	23/22	25/20	N/A	20/8/1/16	N/A	N/A	N/A
				DDLT	55	53.4 ± 9.1^a^	42/13	N/A	N/A	15/27/0/13	19/23/13	25/30	2728	N/A	26/9/2/18	N/A	N/A	N/A
Fisher RA (2007) USA [25]	Retrospective cohort	1998–2003	9	LDLT	58	54.6 ± 9^a^	45/13	95	4	N/A	N/A	21/37	28/28	43/9/3	N/A	N/A	N/A	26/32
				DDLT	34	52.1 ± 10^a^	25/9	353	3.4	N/A	N/A	20/14	24/10	27/3/0	N/A	N/A	N/A	14/20
Terrault NA (2007) USA [26]	Retrospective cohort	1998–2003	9	LDLT	36	N/A	N/A	N/A	N/A	N/A	N/A	N/A	N/A	N/A	N/A	N/A	N/A	N/A
				DDLT	27	N/A	N/A	N/A	N/A	N/A	N/A	N/A	N/A	N/A	N/A	N/A	N/A	N/A
Allam N (2008) KSA [27]	Retrospective cohort	2001–2007	1	LDLT	8	55.14 ± 8.1^a^	6/3	N/A	N/A	N/A	N/A	N/A	N/A	N/A	N/A	N/A	N/A	N/A
				DDLT	15	48.78 ± 17.5^a^	10/4	N/A	N/A	N/A	N/A	N/A	N/A	N/A	N/A	N/A	N/A	N/A
Di Sandro S (2009) Italy [28]	Retrospective cohort	2000–2007	1	LDLT	25	N/A	N/A	107 (11–385)^b^	N/A	N/A	N/A	15/10	N/A	N/A	N/A	N/A	N/A	21/4
				DDLT	154	N/A	N/A	404 (3–1704)^b^	N/A	N/A	N/A	106/48	N/A	N/A	N/A	N/A	N/A	107/47
Vakili K (2009) USA [29]	Retrospective cohort	1999–2007	1	LDLT	28	56 (47–67)^b^	21/7	N/A	3.4 (0.25–8.7)^b^	15/2/0/11	N/A	21/7	26/2	N/A	18/7/2/1	3.4 ± 1.0^a^	6/19/3	5/23
				DDLT	74	N/A	N/A	N/A	N/A	N/A	N/A	N/A	N/A	N/A	N/A	N/A	N/A	N/A
Giacomoni A (2009) Italy [30]	Retrospective cohort	2000–2007	1	LDLT	25	57	N/A	264	N/A	N/A	N/A	15/10	N/A	N/A	N/A	N/A	N/A	21//4
				DDLT	154	54	N/A	404	N/A	N/A	N/A	107/47	N/A	N/A	N/A	N/A	N/A	107/47
Hsieh TH (2010) USA [31]	Retrospective cohort	1999–2008	1	LDLT	15	56	N/A	N/A	N/A	N/A	N/A	11//4	N/A	N/A	N/A	N/A	N/A	4/11
				DDLT	121	56	N/A	N/A	N/A	N/A	N/A	90/31	N/A	N/A	N/A	N/A	N/A	88/33
Sharr WW (2011) China [32]	Retrospective cohort	1995–2005	1	LDLT	90	N/A	N/A	N/A	N/A	N/A	N/A	N/A	90/0	N/A	N/A	N/A	N/A	N/A
				DDLT	34	N/A	N/A	N/A	N/A	N/A	N/A	N/A	34/0	N/A	N/A	N/A	N/A	N/A
Kornberg A (2011) Germany [33]	Retrospective cohort	N/A	1	LDLT	12	N/A	N/A	120	N/A	N/A	N/A	6/6	N/A	N/A	N/A	N/A	N/A	N/A
				DDLT	78	N/A	N/A	365	N/A	N/A	N/A	51/27	N/A	N/A	N/A	N/A	N/A	N/A
Berg CL (2011) USA [34]	Retrospective cohort	2002–2009	9	LDLT	49	N/A	N/A	50.08	N/A	N/A	N/A	N/A	N/A	N/A	N/A	N/A	N/A	N/A
				DDLT	65	N/A	N/A	72.65	N/A	N/A	N/A	N/A	N/A	N/A	N/A	N/A	N/A	N/A
Bhangui P (2011) France [35]	Retrospective cohort	2000–2009	1	LDLT	36	54 ± 7^a^	32/4	78 ± 72^a^	4.8 ± 3^a^	N/A	N/A	26/10	31/5	N/A	N/A	2.9 ± 1.1^a^	N/A	12/24
				DDLT	120	56 ± 8^a^	100/20	237 ± 270^a^	4.2 ± 2.6^a^	N/A	N/A	94/26	104/16	N/A	N/A	3 ± 2.3^a^	N/A	45/75
Azzam AZ (2011) KSA [36]	Retrospective cohort	2001–2011	1	LDLT	18	N/A	N/A	N/A	N/A	N/A	N/A	18/0	N/A	N/A	N/A	N/A	N/A	N/A
				DDLT	34	N/A	N/A	N/A	N/A	N/A	N/A	34/0	N/A	N/A	N/A	N/A	N/A	N/A
Kulik LM (2012) USA [37]	Retrospective cohort	1998–2009	9	LDLT	100	55.2 ± 8^a^	75/25	77.7 ± 106^a^	5.9	78 (HCV)	N/A	41/59	65/35	N/A	2.4 ± 1.8^a^	4.3 ± 2.5^a^	N/A	59/41
				DDLT	97	53.9 ± 8.5^a^	76/21	180.5 ± 258^a^	4.3	78 (HCV)	N/A	71/26	83/14	N/A	2.1 ± 1.7^a^	3.5 ± 1.9^a^	N/A	73/24
Sandhu L (2012) Canada [38]	Retrospective cohort	1996–2009	1	LDLT	58	54.5 ± 8.8^a^	46/12	93 (6–753)^b^	2.5 (0.2–7.3)^b^	8/38/0/12	N/A	42/16	N/A	N/A	1 (0–11)	3.9 (0.5–22)^b^	49 W&M/3 p	29/29
				DDLT	287	55.8 ± 7.1^a^	246/41	159 (0–1071)^b^	3.2 (0–13.4)^b^	81/137/0/69	N/A	189/91	N/A	N/A	1 (0–12)	3.8 (0.5–15.4)^b^	199 W&M/33 p	159/128
Li C (2013) China [39]	Retrospective cohort	2004–2012	1	LDLT	60	45.23 ± 8.18^a^	54/6	N/A	N/A	N/A	N/A	N/A	N/A	35/25/0	N/A	N/A	7/41/12	N/A
				DDLT	156	47.99 ± 9.60^a^	138/18	N/A	N/A	N/A	N/A	N/A	N/A	111/45/0	N/A	N/A	25/106/25	N/A
Lei J (2013) China [40]	Retrospective cohort	2002–2009	1	LDLT	31	44.4 ± 9.7^a^	18/13	N/A	N/A	26/1/1/3	15/3/3	N/A	N/A	N/A	14/8/9	N/A	10/15/8	31/0
				DDLT	52	44.0 ± 8.21^a^	31/21	N/A	N/A	39/3/3/7	14/6/3	N/A	N/A	N/A	22/16/14	N/A	20/20/12	52/0
Xiao GQ (2014) China [41]	Retrospective cohort	1999–2012	1	LDLT	84	44.3	6/78	27	N/A	N/A	43/34/7	22/58	28//52	N/A	44.3	N/A	N/A	17/69
				DDLT	276	47.3	29/247	48	N/A	N/A	137/119/20	69/199	84/184	N/A	47.3	N/A	N/A	101/175
Chen J (2014) China [42]	Retrospective cohort	2007–2010	1	LDLT	47	N/A	44/3	22	N/A	N/A	N/A	N/A	N/A	N/A	N/A	N/A	30 W&M/17 p	N/A
				DDLT	94	N/A	88/6	91	N/A	N/A	N/A	N/A	N/A	N/A	N/A	N/A	55 W&M/39 p	N/A
Park MS (2014) Korea [12]	Retrospective cohort	N/A	1	LDLT	166	52.5 ± 7.7^a^	131/35	N/A	N/A	146/12/0/8	N/A	N/A	N/A	N/A	1.4 ± 0.6^a^	N/A	N/A	96/70
				DDLT	50	54.3 ± 9.6^a^	29/21	N/A	N/A	39/6/0/5	N/A	N/A	N/A	N/A	1.5 ± 0.7^a^	N/A	N/A	33/17
Wan P (2014) China [43]	Retrospective cohort	2007–2010	1	LDLT	40	48.6 ± 9.7^a^	N/A	N/A	1.75 (0.08–6.25)^b^	40/0/0/0	12/18/10	24/16	N/A	N/A	48.6 ± 9.7^a^	N/A	N/A	12/28
				DDLT	80	49.5 ± 8.9^a^	N/A	N/A	24 (0.08–6.3)^b^	76/0/0/4	25/38/17	48/32	N/A	N/A	49.5 ± 8.9^a^	N/A	N/A	29/51
Bonadio I (2014) Belgium [44]	Retrospective cohort	2000–2007	1	LDLT	28	N/A	N/A	N/A	N/A	N/A	N/A	N/A	N/A	N/A	N/A	N/A	N/A	N/A
				DDLT	48	N/A	N/A	N/A	N/A	N/A	N/A	N/A	N/A	N/A	N/A	N/A	N/A	N/A
Ninomiya M (2015) Japan/USA [14]	Retrospective cohort	2002–2010	2	LDLT	133	57.6 ± 7.1^a^	78/55	44 (4–236)^b^	6.3	21/100/12	N/A	N/A	N/A	N/A	4.8 ± 7.9^a^	2.4 ± 1.1^a^	7.5%/63.9%/28.6%	N/A
				DDLT	362	58.3 ± 7.4^a^	285/77	196 (0–3996)^b^	5.6	51/212/99	N/A	N/A	N/A	N/A	2.6 ± 2.2^a^	2.8 ± 1.8^a^	40.8%/49.7%/9.7%	N/A
Chen LP (2015) China [45]	Retrospective cohort	2005–2013	1	LDLT	66	45.82 ± 7.72^a^	60//6	23.37 ± 16.32	N/A	N/A	N/A	34/32	42/24	N/A	N/A	5.22 ± 2.31^a^	13/45/8	N/A
				DDLT	163	47.93 ± 9.51^a^	144/19	46.88 ± 32.12^a^	N/A	N/A	N/A	72/91	95/68	N/A	N/A	5.24 ± 2.24^a^	27/110/26	N/A
Tomiyama K (2016) Canada [46]	Retrospective cohort	2000–2004	1	LDLT	106	N/A	N/A	264 (189–450)^b^	N/A	N/A	N/A	N/A	N/A	N/A	N/A	N/A	N/A	N/A
				DDLT	434	N/A	N/A	465 (210–891)^b^	N/A	N/A	N/A	N/A	N/A	N/A	N/A	N/A	N/A	N/A
Fonseca E (2016) Brazil [47]	Retrospective cohort	2000–2009	1	LDLT	43	N/A	N/A	N/A	N/A	N/A	N/A	N/A	N/A	N/A	N/A	N/A	N/A	N/A
				DDLT	23	N/A	N/A	N/A	N/A	N/A	N/A	N/A	N/A	N/A	N/A	N/A	N/A	N/A
Azoulay D (2017) France [48]	Retrospective cohort	2000–2009	5	LDLT	75	54.2 ± 7.6^a^	62/13	78 ± 69^a^	8.5 ± 1.9^a^	N/A	46 A&B/ 28 C	44/31	N/A	N/A	N/A	N/A	N/A	53/22
				DDLT	576	56.3 ± 7.4^a^	499/77	183 ± 219^a^	5.6 ± 13.4^a^	N/A	441/76	333/243	N/A	N/A	N/A	N/A	N/A	366/205
Goldaracena N (2019) Canada [11]	Retrospective cohort	2000–2015	1	LDLT	118	N/A	N/A	108 (75–195)^b^	4 (1.7–8.2)^b^	N/A	N/A	N/A	N/A	N/A	N/A	N/A	N/A	64/54
				DDLT	527	N/A	N/A	189 (96–336)^b^	4.3 (2–9)^b^	N/A	N/A	N/A	N/A	N/A	N/A	N/A	N/A	380/147
Wong TCL (2019) China [10]	Retrospective cohort	1995–2014	1	LDLT	161	55 (30–73)^b^	129/32	N/A	N/A	125 (HBV) 28 (HCV)	81/48/32	113/48	141/20	N/A	85/47/20	2.9 (0.90–8.80)^b^	45/91/10	N/A
				DDLT	85	57 (41–68)^b^	72/13	N/A	N/A	76 (HBV) 6 (HCV)	32/31/22	72/13	85/0	N/A	57/17/11	2.4 (0.70–0.60)^b^	16/46/6	N/A
Lee S (2020) Korea [49]	Retrospective cohort	2005–2015	1	LDLT	829	53.7 ± 6.2^a^	709/120	N/A	N/A	728 (HBV) 45 (HCV)	346/319/164	N/A	N/A	N/A	N/A	1.5 ± 1.6^a^	N/A	635/194
				DDLT	67	54.7 ± 7.7^a^	51/16	N/A	N/A	58 (HBV) 4 (HCV)	8/20/39	N/A	N/A	N/A	N/A	1.7 ± 2.0^a^	N/A	53/14
Rahatli S (2020) Turkey [50]	Retrospective cohort	1988–2018	1	LDLT	29	N/A	N/A	N/A	N/A	N/A	N/A	N/A	N/A	N/A	N/A	N/A	N/A	N/A
				DDLT	20	N/A	N/A	N/A	N/A	N/A	N/A	N/A	N/A	N/A	N/A	N/A	N/A	N/A

^a^The results are presented as means and standard deviation

^b^The results are presented as median and range

**Table 2:** Newcastle–Ottawa scale for included studies

Study	Representativeness of expose of cohort	Selection of the non-exposed cohort	Ascertainment of exposure	Demonstration that outcome of interest was not present at start of study	Comparability	Assessment of outcome	Adequate follow-up length	Adequacy of followup	Score
Gondolesi GE (2004) USA	*	*	*	*	**	*	*	*	9
Roayaie S (2004) USA	*	*	*	*		*	*	*	7
Hwang S (2005) Korea	*	*	*	*	**	*	*	*	9
Karakayali H (2006) Turkey	*	*	*	*	**	*			7
Sotiropoulos GC (2007) Germany	*	*	*	*	**	*	*	*	9
Fisher RA (2007) USA	*	*	*	*	**	*	*	*	9
Terrault NA (2007) USA	*	*	*	*	**	*			7
Allam N (2008) KSA	*	*	*	*	*	*	*	*	8
Di Sandro S (2009) Italy	*	*	*	*	*	*	*	*	9
Vakili K (2009) USA	*	*	*	*		*	*	*	7
Berg CL (2011) USA	*	*	*	*	*	*	*	*	8
Bhangui P (2011) France	*	*	*	*	**	*	*	*	9
Azzam AZ (2011) KSA	*	*	*	*	*	*	*		7
Kulik LM (2012) USA	*	*	*	*	**	*	*	*	9
Sandhu L (2012) Canada	*	*	*	*	**	*	*	*	9
Li C (2013) China	*	*	*	*	**	*	*	*	9
Lei J (2013) China	*	*	*	*	**	*	*	*	9
Xiao GQ (2014) China	*	*	*	*	*	*	*		7
Chen J (2014) China	*	*	*	*	*	*	*		7
Park MS (2014) Korea	*	*	*	*	**	*	*	*	9
Wan P (2014) China	*	*	*	*	**	*	*	*	9
Bonadio I (2014) Belgium	*	*	*	*	*	*	*		7
Ninomiya M (2015) Japan USA	*	*	*	*	*	*	*	*	8
Chen LP (2015) China	*	*	*	*	**	*	*		8
Azoulay D (2017) France	*	*	*	*	**	*	*	*	9
Goldaracena N (2019) Canada	*	*	*	*	*	*	*	*	8
Wong TCL (2019) China	*	*	*	*	**	*	*	*	9
Lee S (2020) Korea	*	*	*	*	**	*	*	*	9
Rahatli S (2020) Turkey	*	*	*	*		*	*	*	7

* stands for one point

** stands for two points

### Primary outcome

#### Overall survival

**Fig. 2:**
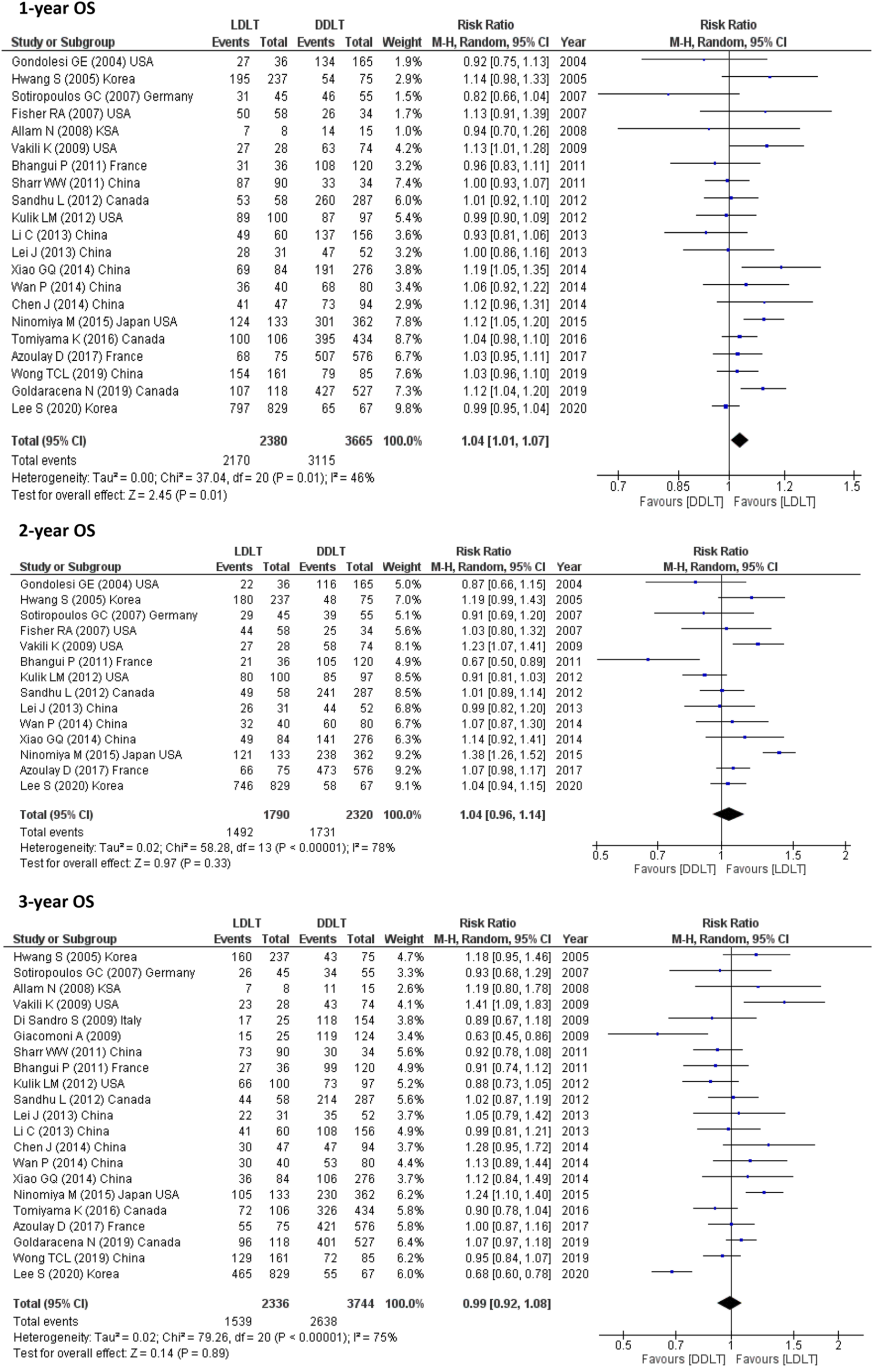

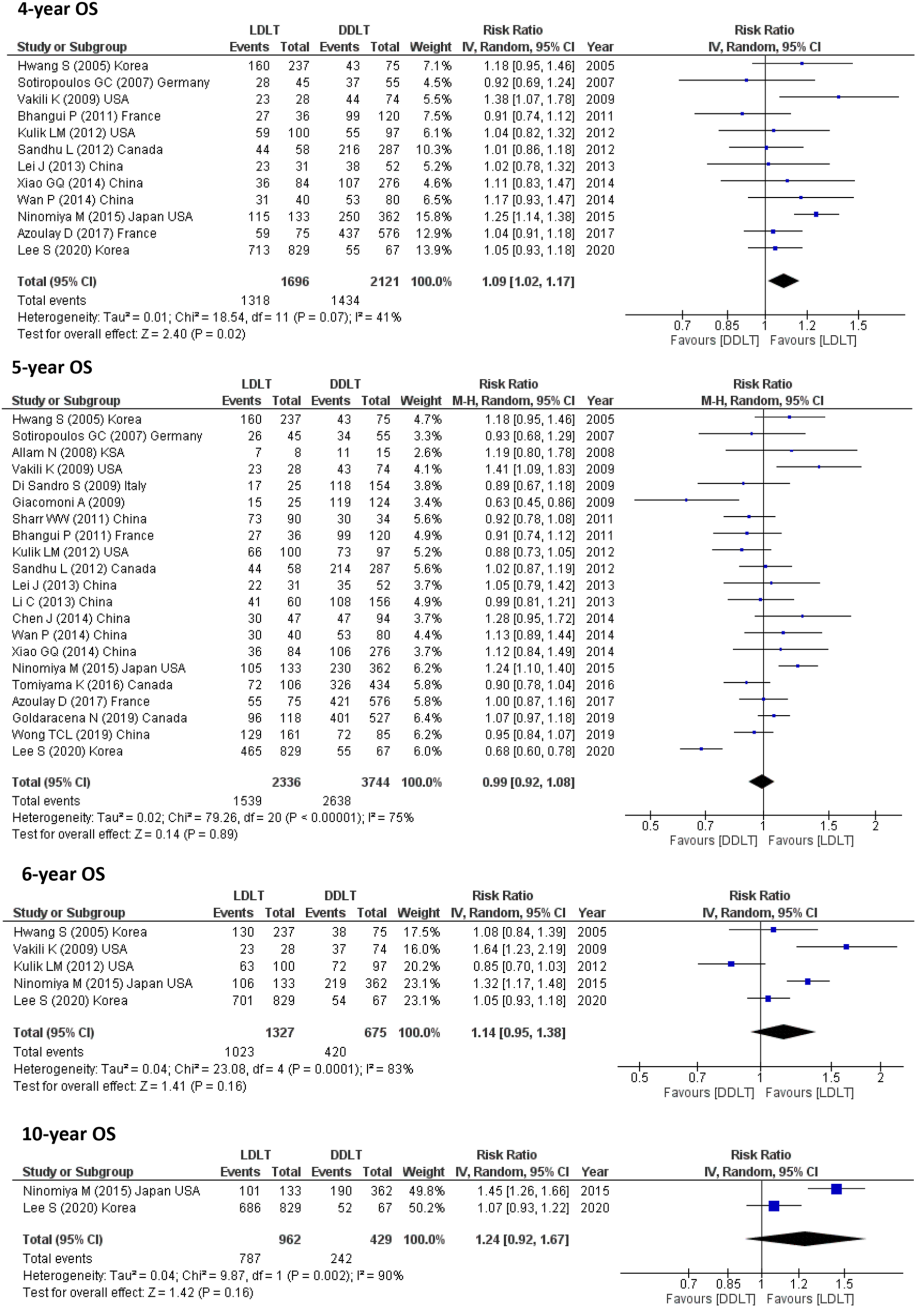
OS for LDLT and DDLT recipients

21 studies (6045 participants) assessed 1-year OS, 19 studies (5859) reported 3-year OS and 12 studies (3817) calculated 4 year-OS. The pooled results from these studies showed possible improvement for LDLT recipients as follows (1-year OS, RR = 1.04, 95% CI 1.01–1.07, *p* = 0.01; *I*^2^ = 46%) and (3-year OS, RR = 1.07, 95% CI 1.01–1.13, *p* = 0.02; *I*^2^ = 63%) However, this meta-analysis showed that LDLT recipients and DDLT recipients had similar 5-, 6- and 10-year OS (5-year OS, RR = 0.99, 95% CI 0.92–1.08, *p* = 0.89; *I*^2^ = 75%) and (10-year OS, RR = 1.24, 95% CI 0.92–1.67, *p* = 0.16; *I*^2^ = 90%) as shown in 21 studies (6080) for 5-year OS, 5 studies (2002) for 6-year OS and 2 studies (1391) for 10-year OS (Fig. 2).

#### RFS

**Fig. 3:**
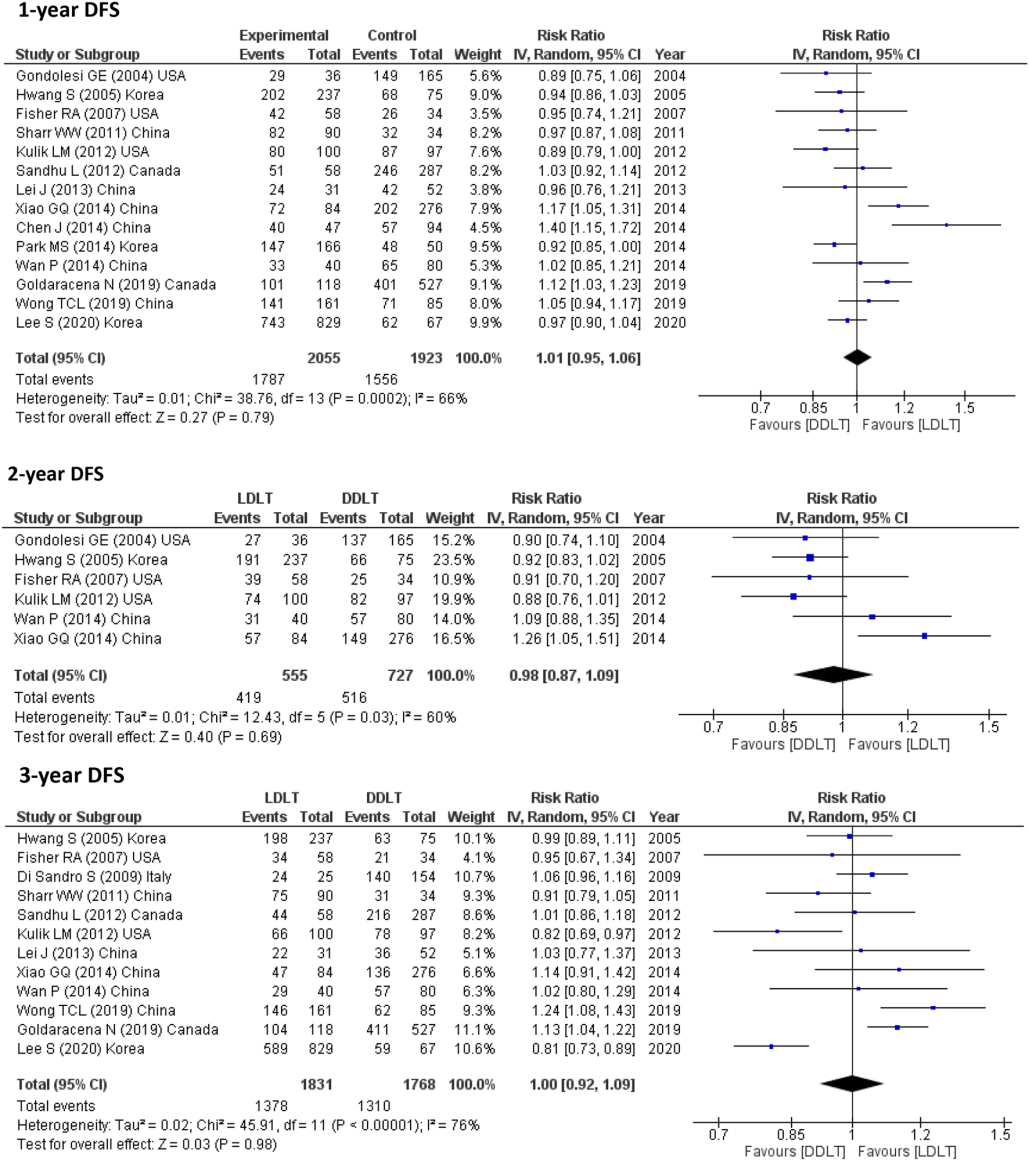

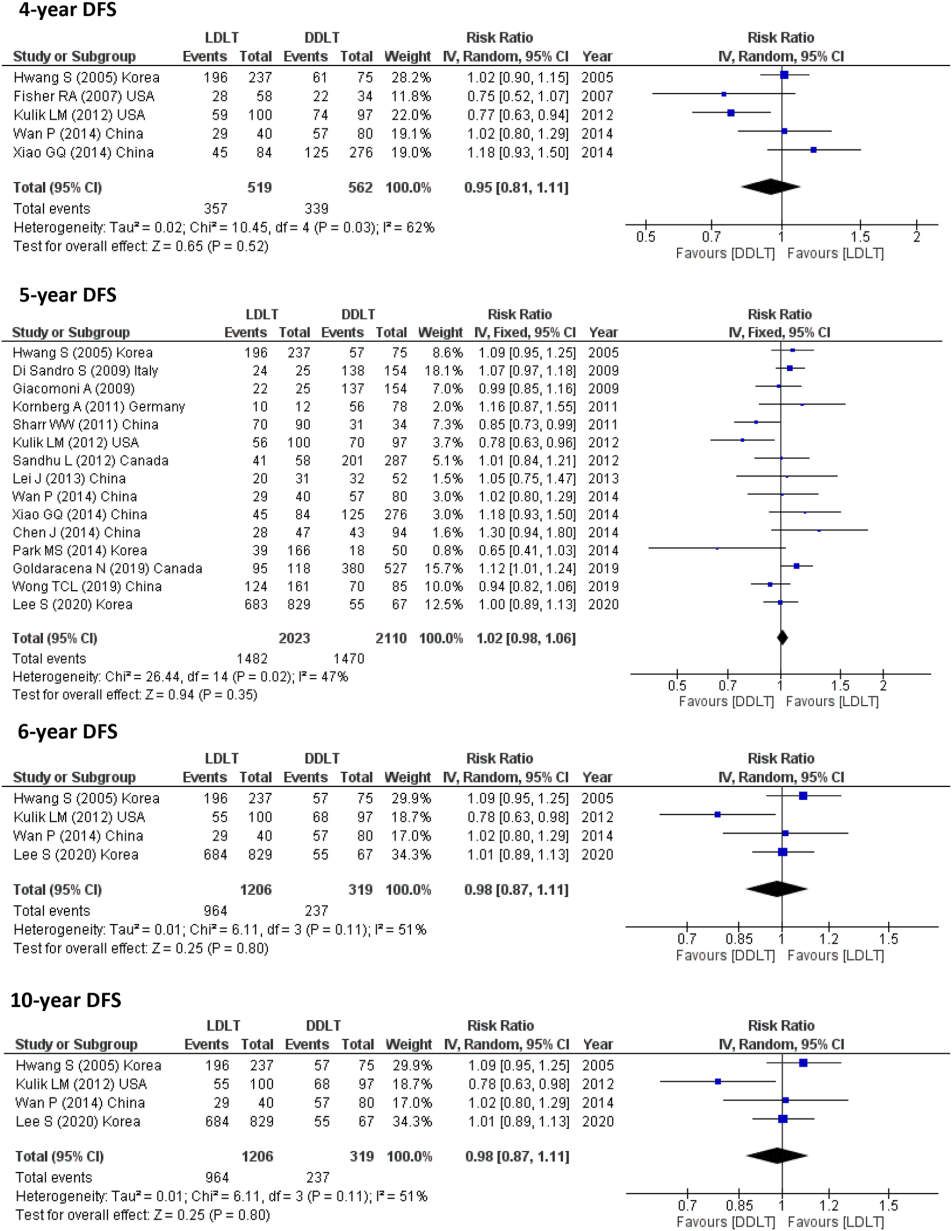
DFS for LDLT and DDLT recipients

14 studies (3978 participants) reported 1-year RFS, 6 studies (1282 participants) assessed 2-year DFS, 12 studies (3599 participants) reported 3-year RFS, 5 studies (1081 participants) calculated 4-year DFS, 15 studies (4133 participants) assessed 5-year DFS, 4 studies (1525 participants) reported 6-year DFS and only one study assessed 10-year DFS (896 participants). The pooled results from these studies showed no significant difference between LDLT and DDLT (Fig. 3).

#### ITT-OS

**Fig. 4:**
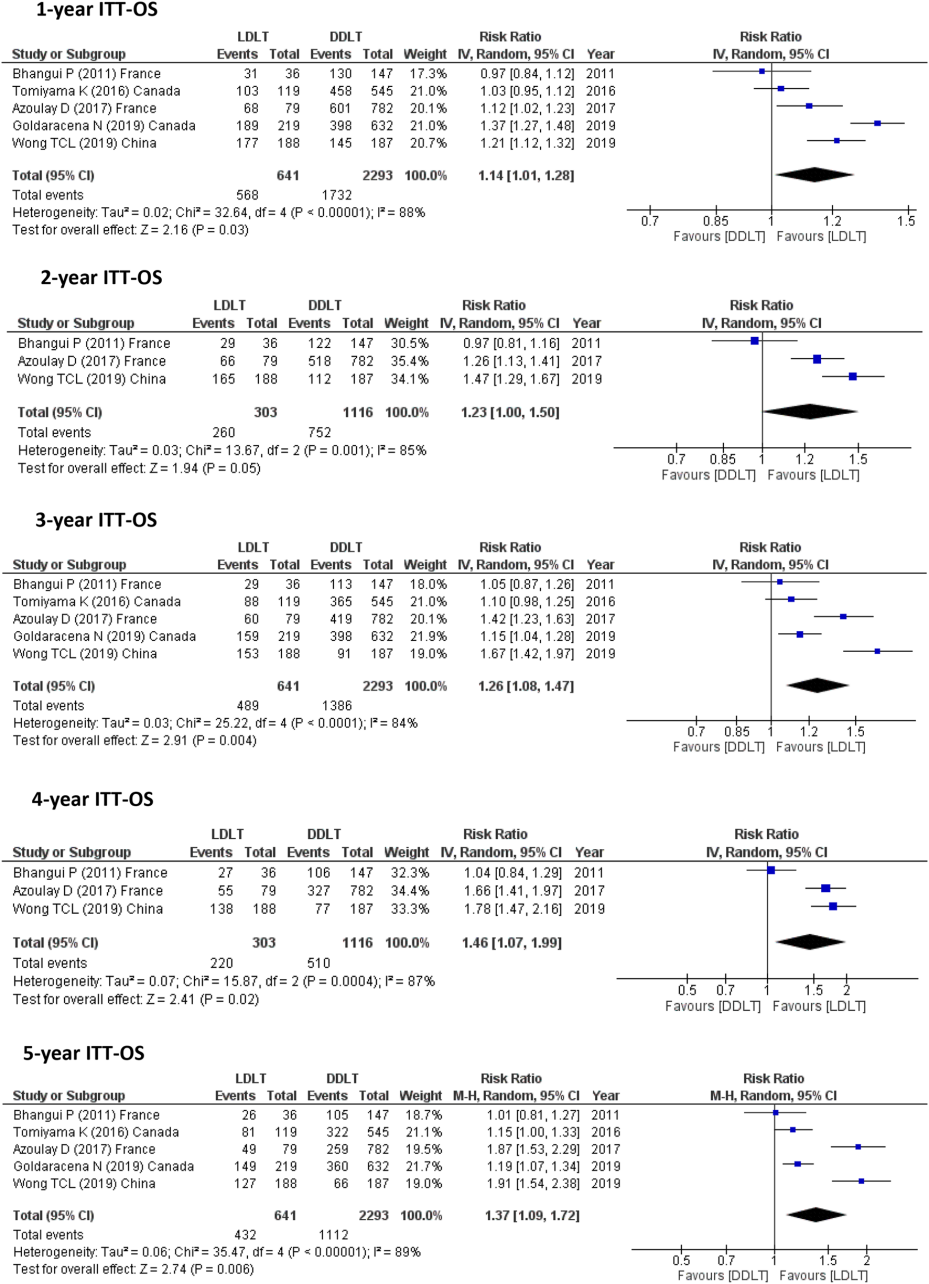
ITT-OS for LDLT and DDLT recipients

While 1-, 3- and 5-year ITT-OS were reported in 5 studies (2934 participants) and 2-, 4-year ITT-OS were assessed in 3 studies (1419), no study calculated the 6-, 10-year ITT-OS. Significant improvement was observed for LDLT recipients especially for 5-year ITT-OS. (1-year, RR = 1.14, 95% CI 1.01–1.28, *P* = 0.03; *I*^2^ = 88%), (2-year, RR = 1.23, 95% CI 1.00–1.50, *P* = 0.05; *I*^2^ = 85%), (3-year, RR = 1.26, 95% CI 1.08–1.47, *P* = 0.004; *I*^2^ = 84%), (4-year, RR = 1.46, 95% CI 1.07–1.99, *P* = 0.02; *I*^2^ = 87%) and (5-year, RR = 1.37, 95% CI 1.09–1.72, *P* = 0.006; *I*^2^ = 89%) (Fig. 4).

#### Recurrence rates

**Fig. 5:**
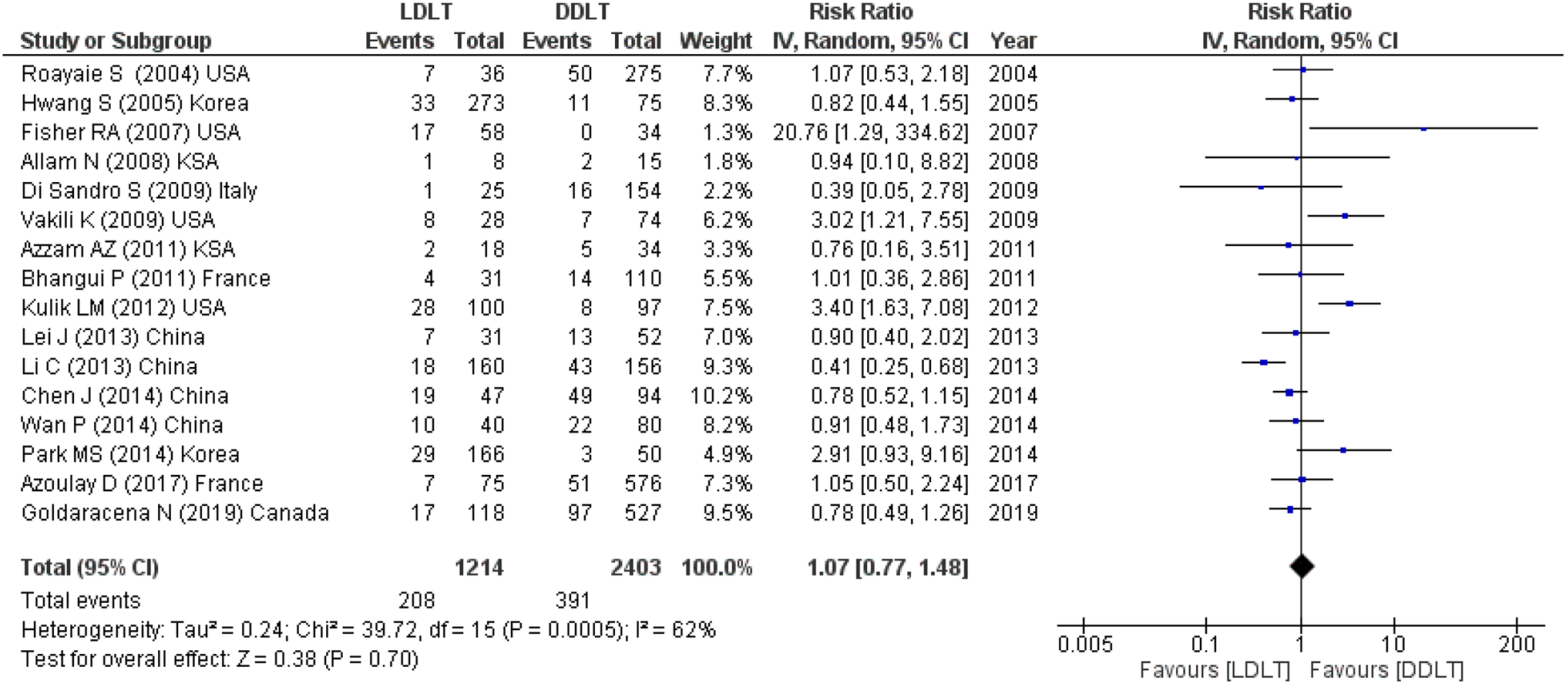
Recurrence for LDLT and DDLT recipients

The number of HCC recurrence was pooled from 16 studies (3617 participants) and showed comparable recurrence between recipients after LDLT and DDLT (RR = 1.07, 95% CI 0.77–1.48, *P* = 0.70; *I*^2^ = 62%) (Fig. 5).

#### Subgroup analysis

To investigate the source of heterogeneity among studies, a subgroup analysis was carried out by stratifying the analysis according to Milan criteria and region of transplant.

#### Milan criteria

We performed an additional comparative analysis of LDLT and DDLT in patients with HCC meeting or exceeding the Milan criteria regarding 0S and DFS (Table 3). For those meeting the Milan criteria, no significant difference in OS and DFS could be detected between LDLT and DDLT recipients. On the other hand, OS for those exceeding Milan criteria was better after LDLT. However, there were insufficient data to detect DFS for patients beyond Milan criteria. Notably, the outcome for those exceeding Milan criteria should be carefully interpreted because of the limited data (Tables 3, 4).

**Table 3:** OS for LDLT and DDLT within and beyond Milan criteria

Subgroup	Outcome	Studies (*n*)	Patient (*n*)	Effect estimate [RR (95% CI)]	Heterogeneity	Test for overall effect	Favour group
Within Milan	1 year OS	5	1593	1.04 [0.96, 1.12]	*I*^2^ = 72% (*p* = 0.006)	*Z* = 1.02 (*p* = 0.31)	None
	2 year OS	4	1502	1.06 [0.97, 1.16]	*I*^2^ = 70% (*p* = 0.02)	*Z* = 1.23 (*p* = 0.22)	None
	3 year OS	5	1580	1.01 [0.88, 1.16]	*I*^2^ = 81% (*p* = 0.0002)	*Z* = 0.15 (*p* = 0.88)	None
	4 year OS	4	1502	1.07 [0.92, 1.25]	*I*^2^ = 83% (*p* = 0.0005)	*Z* = 0.85 (*p* = 0.39)	None
	5 year OS	5	1593	1.10 [0.93, 1.29]	*I*^2^ = 83% (*p* = 0.0001)	*Z* = 1.10 (*p* = 0.27)	None
	6 year OS	3	1271	1.22 [0.97, 1.52]	*I*^2^ = 88% (*p* = 0.0002)	*Z* = 1.70 (*p* = 0.09)	None
	10 year OS	2	1078	1.23 [0.83, 1.84]	*I*^2^ = 96% (*p* < 0.00001)	*Z* = 1.04 (*p* = 0.30)	None
Beyond Milan	1 year OS	4	501	1.02 [0.94, 1.10]	*I*^2^ = 0% (*p* = 0.73)	*Z* = 0.50 (*p* = 0.62)	None
	2 year OS	4	501	1.06 [0.95, 1.18]	*I*^2^ = 0% (*p* = 0.83)	*Z* = 0.98 (*p* = 0.33)	None
	3 year OS	4	501	1.16 [1.01, 1.32]	*I*^2^ = 0% (*p* = 0.60)	*Z* = 2.16 (*p* = 0.03)	LDLT
	4 year OS	4	501	1.20 [1.04, 1.38]	*I*^2^ = 32% (*p* = 0.22)	*Z* = 2.44 (*p* = 0.01)	LDLT
	5 year OS	3	420	1.32 [1.13, 1.54]	*I*^2^ = 0% (*p* = 0.79)	*Z* = 3.44 (*p* = 0.0006)	LDLT
	6 year OS	2	313	1.30 [1.03, 1.64]	*I*^2^ = 0% (*p* = 0.75)	*Z* = 2.25 (*p* = 0.02)	LDLT
	10 year OS	2	313	1.42 [1.07, 1.87]	*I*^2^ = 34% (*p* = 0.22)	*Z* = 2.47 (*p* = 0.01)	LDLT

**Table 4:** DFS for LDLT and DDLT within and beyond Milan criteria

Subgroup	Outcome	Studies (*n*)	Patient (*n*)	Effect estimate [RR (95% CI)]	Heterogeneity	Test for overall effect	Favour group
Within Milan	1 year DFS	2	853	0.99 [0.89, 1.10]	*I*^2^ = 64% (*p* = 0.10)	*Z* = 0.18 (*p* = 0.86)	None
	2 year DFS	1	762	N/A	N/A	N/A	N/A
	3 year DFS	2	853	0.93 [0.89, 0.97]	*I*^2^ = 6% (*p* = 0.30)	*Z* = 3.42 (*p* = 0.0006)	DDLT
	4 year DFS	1	762	N/A	N/A	N/A	N/A
	5 year DFS	2	853	0.96 [0.83, 1.11]	*I*^2^ = 47% (*p* = 0.17)	*Z* = 0.61 (*p* = 0.54)	None
	6 year DFS	1	762	N/A	N/A	N/A	N/A
	10 year DFS	1	762	N/A	N/A	N/A	N/A
Beyond Milan	1 year DFS	1	134	N/A	N/A	N/A	N/A
	2 year DFS	1	134	N/A	N/A	N/A	N/A
	3 year DFS	1	134	N/A	N/A	N/A	N/A
	4 year DFS	1	134	N/A	N/A	N/A	N/A
	5 year DFS	1	134	N/A	N/A	N/A	N/A
	6 year DFS	1	134	N/A	N/A	N/A	N/A
	10 year DFS	1	134	N/A	N/A	N/A	N/A

#### Region (Asia, America and Europe)

Another comparison was done to detect the OS and DFS between LDLT and DDLT recipients according to the region of transplant (Asia, America and Europe). No remarkable difference in OS between LDLT and DDLT could be detected according to the region (Tables 5, 6).

**Table 5:** OS for LDLT and DDLT according to the region of transplantation

Subgroup	Outcome	Studies (*n*)	Patient (*n*)	Effect estimate [RR (95% CI)]	Heterogeneity	Test for overall effect	Favour group
Asia	1 year OS	11	2722	1.03 [0.98, 1.07]	*I*^2^ = 44% (*p* = 0.06)	*Z* = 1.14 (*p* = 0.25)	None
	2 year OS	5	1771	1.07 [1.00, 1.14]	*I*^2^ = 0% (*p* = 0.56)	*Z* = 1.82 (*p* = 0.07)	None
	3 year OS	8	2357	1.09 [0.98, 1.21]	*I*^2^ = 68% (*p* = 0.003)	*Z* = 1.64 (*p* = 0.10)	None
	4 year OS	5	1771	1.08 [1.00, 1.18]	*I*^2^ = 0% (*p* = 0.81)	*Z* = 1.89 (*p* = 0.06)	None
	5 year OS	9	1625	1.03 [0.95, 1.12]	*I*^2^ = 22% (*p* = 0.25)	*Z* = 0.82 (*p* = 0.41)	None
	6 year OS	2	1208	1.06 [0.95, 1.18]	*I*^2^ = 0% (*p* = 0.83)	*Z* = 0.97 (*p* = 0.33)	None
	10 year OS	1	896	N/A	N/A	N/A	N/A
America	1 year OS	8	2434	1.06 [1.02, 1.10]	*I*^2^ = 31% (*p* = 0.18)	*Z* = 3.08 (*p* = 0.002)	LDLT
	2 year OS	5	937	1.01 [0.89, 1.15]	*I*^2^ = 67% (*p* = 0.02)	*Z* = 0.21 (*p* = 0.83)	None
	3 year OS	6	1921	1.05 [0.93, 1.18]	*I*^2^ = 77% (*p* = 0.0005)	*Z* = 0.75 (*p* = 0.45)	None
	4 year OS	3	644	1.11 [0.93, 1.34]	*I*^2^ = 54% (*p* = 0.11)	*Z* = 1.13 (*p* = 0.26)	None
	5 year OS	5	1829	1.02 [0.90, 1.16]	*I*^2^ = 68% (*p* = 0.01)	*Z* = 0.31 (*p* = 0.76)	None
	6 year OS	2	299	1.17 [0.61, 2.23]	*I*^2^ = 93% (*p* = 0.0002)	*Z* = 0.48 (*p* = 0.63)	None
	10 year OS	0	0	N/A	N/A	N/A	N/A
Europe	1 year OS	5	1420	0.99 [0.91, 1.09]	*I*^2^ = 44% (*p* = 0.13)	*Z* = 0.13 (*p* = 0.90)	None
	2 year OS	3	907	0.88 [0.64, 1.22]	*I*^2^ = 85%(*p* = 0.001)	*Z* = 0.77 (*p* = 0.44)	None
	3 year OS	4	1086	1.03 [0.95, 1.13]	*I*^2^ = 0% (*p* = 0.72)	*Z* = 0.75 (*p* = 0.46)	None
	4 year OS	3	907	0.99 [0.90, 1.10]	*I*^2^ = 0% (*p* = 0.50)	*Z* = 0.18 (*p* = 0.86)	None
	5 year OS	4	1079	0.87 [0.71, 1.06]	*I*^2^ = 58% (*p* = 0.07)	*Z* = 1.35 (*p* = 0.18)	None
	6 year OS	1	197	N/A	N/A	N/A	N/A
	10 year OS	0	0	N/A	N/A	N/A	N/A

**Table 6:** DFS for LDLT and DDLT according to the region of transplantation

Subgroup	Outcome	Studies (*n*)	Patient (*n*)	Effect estimate [RR (95% CI)]	Heterogeneity	Test for overall effect	Favour group
Asia	1 year DFS	9	2498	1.02 [0.95, 1.09]	*I*^2^ = 69% (*p* = 0.001)	*Z* = 0.52 (*p* = 0.60)	None
	2 year DFS	3	792	1.07 [0.87, 1.31]	*I*^2^ = 78% (*p* = 0.010)	*Z* = 0.61 (*p* = 0.54)	None
	3 year DFS	7	2141	1.00 [0.88, 1.14]	*I*^2^ = 79% (*p* < 0.0001)	*Z* = 0.03 (*p* = 0.98)	None
	4 year DFS	3	792	1.04 [0.95, 1.15]	*I*^2^ = 0% (*p* = 0.53)	*Z* = 0.85 (*p* = 0.39)	None
	5 year DFS	10	2843	1.00 [0.93, 1.08]	*I*^2^ = 38% (*p* = 0.11)	*Z* = 0.00 (*p* = 1.00)	None
	6 year DFS	3	1328	1.04 [0.95, 1.13]	*I*^2^ = 0% (*p* = 0.68)	*Z* = 0.82 (*p* = 0.41)	None
	10 year DFS	1	896	N/A	N/A	N/A	N/A
America	1 year DFS	5	1480	0.99 [0.89, 1.09]	*I*^2^ = 68% (*p* = 0.01)	*Z* = 0.28 (*p* = 0.78)	None
	2 year DFS	3	490	0.89 [0.80, 0.99]	*I*^2^ = 0% (*p* = 0.95)	*Z* = 2.14 (*p* = 0.03)	DDLT
	3 year DFS	4	1279	0.99 [0.84, 1.16]	*I*^2^ = 74% (*p* = 0.008)	*Z* = 0.18 (*p* = 0.85)	None
	4 year DFS	2	289	0.77 [0.64, 0.91]	*I*^2^ = 0% (*p* = 0.86)	*Z* = 2.99 (*p* = 0.003)	DDLT
	5 year DFS	3	1187	0.97 [0.79, 1.19]	*I*^2^ = 78% (*p* = 0.01)	*Z* = 0.27 (*p* = 0.78)	None
	6 year DFS	1	197	N/A	N/A	N/A	N/A
	10 year DFS	0	0	N/A	N/A	N/A	N/A
Europe	1 year DFS	0	0	N/A	N/A	N/A	N/A
	2 year DFS	0	0	N/A	N/A	N/A	N/A
	3 year DFS	1	179	N/A	N/A	N/A	N/A
	4 year DFS	0	0	N/A	N/A	N/A	N/A
	5 year DFS	3	448	1.06 [0.98, 1.14]	*I*^2^ = 0% (*p* = 0.55)	*Z* = 1.35 (*p* = 0.18)	None
	6 year DFS	0	0	N/A	N/A	N/A	N/A
	10 year DFS	0	0	N/A	N/A	N/A	N/A

### Secondary outcome

A secondary outcome was to detect the prognostic valves and predictors of recurrence after liver transplantation for HCC patients other than the type of graft.

#### Prognostic factors and predictor values of recurrence

The age and the sex of the recipient could not be used as a prognostic factor or as a predictive value of recurrence after liver transplantation. On the other hand, a remarkable decrease in survival and increase in the recurrence rate are associated with tumors which are beyond Milan criteria, number, size of the tumor, high levels of AFP (> 400 ng), the presence of vascular invasion and the poorly differentiated tumors (Tables 7, 8).

**Table 7:** Prognostic factors after liver transplantation

Variable	Studies (*n*)	Effect estimate [HR (95% CI)]	Heterogeneity	Test for overall effect	Reference
Recipient male sex	2	0.97 [0.74, 1.27]	*I*^2^ = 38% (*p* = 0.20)	*Z* = 0.21 (*p* = 0.83)	Female sex
Recipient age, years	2	1.01 [0.99, 1.03]	*I*^2^ = 24% (*p* = 0.27)	*Z* = 1.17 (*p* = 0.24)	Per 1 year increase
Beyond Milan criteria	2	1.89 [1.19, 3.00]	*I*^2^ = 71% (*p* = 0.03)	*Z* = 2.69 (*p* = 0.007)	Within Milan
AFP > 400	0	N/A	N/A	N/A	N/A
No. of tumor nodules	2	1.04 [1.01, 1.07]	*I*^2^ = 70% (*p* = 0.03)	*Z* = 2.40 (*p* = 0.02)	Per 1 nodule increase
Largest tumor diameter, cm	2	1.09 [1.05, 1.12]	*I*^2^ = 33% (*p* = 0.22)	*Z* = 4.91 (*p* < 0.00001)	Per 1 cm increase
Microscopic vascular invasion	2	1.89 [1.52, 2.36]	*I*^2^ = 0% (*p* = 0.44)	*Z* = 5.64 (*p* < 0.00001)	No
Macroscopic vascular invasion	1	N/A	N/A	N/A	N/A
Poor differentiation	2	1.65 [1.21, 2.25]	*I*^2^ = 0% (*p* = 0.69)	*Z* = 3.16 (*p* = 0.002)	Well/mod differentiated

**Table 8:** Predictor values of recurrence after liver transplantation

Variable	Studies (*n*)	Effect estimate [HR (95% CI)]	Heterogeneity	Test for overall effect	Reference
Recipient male sex	3	1.02 [0.70, 1.48]	*I*^2^ = 0% (*p* = 0.83)	*Z* = 0.09 (*p* = 0.93)	Female sex
Recipient age, years	4	0.99 [0.97, 1.01]	*I*^2^ = 0% (*p* = 0.57)	*Z* = 1.12 (*p* = 0.26)	Per 1 year increase
Beyond Milan criteria	4	2.81 [1.69, 4.69]	*I*^2^ = 73% (*p* = 0.005)	*Z* = 3.98 (*p* < 0.0001)	Within Milan
AFP > 400	3	3.70 [2.11, 6.47]	*I*^2^ = 33% (*p* = 0.22)	*Z* = 4.58 (*p* < 0.00001)	AF* P* < 400
No. of tumor nodules	4	1.14 [1.08, 1.20]	*I*^2^ = 46% (*p* = 0.12)	*Z* = 4.83 (*p* < 0.00001)	Per 1 nodule increase
Largest tumor diameter, cm	4	1.19 [1.06, 1.32]	*I*^2^ = 86% (*p* < 0.00001)	*Z* = 3.04 (*p* = 0.002)	Per 1 cm increase
Microscopic vascular invasion	4	3.73 [2.78, 5.01]	*I*^2^ = 26% (*p* = 0.25)	*Z* = 8.77 (*p* < 0.00001)	No
Macroscopic vascular invasion	3	3.88 [2.64, 5.70]	*I*^2^ = 53% (*p* = 0.12)	*Z* = 6.91 (*p* < 0.00001)	No
Poor differentiation	2	2.69 [1.29, 5.61]	*I*^2^ = 62% (*p* = 0.07)	*Z* = 2.63 (*p* = 0.008)	Well/mod differentiated

#### Publication bias assessment

No evidence of publication bias could be detected. The funnel plot analysis showed a symmetrical appearance.

## Discussion

Regarding the overall survival after liver transplantation, while some studies reported equal OS after LDLT and DDLT, some papers reported better survival after LDLT. In our study, pooled patient OS showed trivial improvement in LDLT recipients especially the 3-year OS. However, the long-term OS (5- and 10-year OS) did not show any significant difference between the two types of transplant. In addition to that, according to the pooled results of five studies, our subgroup analysis showed equal long-term OS between LDLT and DDLT for those who are within MC. Nevertheless, beyond Milan criteria, there was a better prognosis, could be detected for the patients who underwent LDLT but this data should be treated cautiously due to the small sample size.

On the other hand, according to a French study, the OS from the time of listing was similar for both LDLT and DDLT. However, this has been explained by a Canadian study by the small sample size that failed to address the better outcome after LDLT. This meta-analysis illustrated that the patients listed for LDLT showed a dramatic increase in the OS (ITT-OS) than those listed for DDLT. This could be attributed to the short waiting time and the low dropout rate. Thus it can be said that if dropout was taken into consideration, LDLT provided much better survival benefits to HCC patients especially in regions that suffer from low deceased organ availability as it provides an endless source of donors and eliminate the probability of progression while waiting [10].

Whether HCC recurrence is more frequent in LDLT remains controversial. Some studies attributed the high levels of recurrence after LDLT in their studies to the growth factors that are released during the natural course of liver regeneration of a partial liver graft [29] and according to Fisher et al. [25], the technique of living donor transplant is the determent factor for recurrence due to greater manipulation of the native liver and preservation of the native vena cava, as well as more hepatic artery and bile duct length that results in leaving residual tumor or violating tumor capsule and tumor embolization through the hepatic veins.

However, in our study, no remarkable difference could be detected between LDLT and DDLT recipients in the DFS. Moreover, our subgroup analysis showed equal DFS between the two groups for those who are within MC. In addition to that no difference in the recurrence rate could be detected between LDLT and DDLT receipts and according to Bhangui et al. [35], there was no difference in the severity of recurrence at presentation in the two groups.

Moreover, the high incidence of recurrence in LDLT recipients that was mentioned in some studies could be explained by two reasons; first, the fast tracking to LDLT may not allow sufficient time for evaluation of the biological aggressiveness of tumors [29, 37]. Secondly, the presence of other factors related to the biology of the tumor, not the graft type. For instance, in the Fisher et al. [25] study, while 15% of the patients in the LDLT group had poorly differentiated tumors, only 3% of DDLT had poorly differentiated tumors and in the study by Vakili et al. [29] 46% of the tumors in the LDLT group had microvascular invasion. In other words, Macrovascular invasion, preoperative serum alpha-fetoprotein (AFP) level, tumor size, histopathologic grading were significant factors for survival and tumor-free survival by univariate analysis [28, 38].

Our secondary outcome was to detect these factors that affect the survival and recurrence after liver transplantation for HCC. To begin with, according to four of the included studies, the age and the sex of the receipt is not considered prognostic factor after transplantation.

Nevertheless, the MC have been well adopted worldwide as a set of guidelines for listing patients for LT [5]. However, these criteria are criticized for being too stringent, since many patients beyond the criteria could still have reasonable post-LT survival [51–53]. Nevertheless, according to the pooled hazard ratio, a significant increase in the recurrence of HCC could be detected for those who were beyond Milan criteria.

In addition to that the biological markers could be used as a predictive value after liver transplantation. In other words, a high AFP level has been shown to be associated with poorer outcomes but the exact consensual cut-off value remains undefined [12, 38]. According to some recent studies, an AFP level of 54 ng/mL was associated with disease recurrence, and AFP level of 105 ng/mL was found to decrease overall survival [15]. In addition to that, using an AFP level > 1000 ng/mL as an exclusion criterion for LT within the MC may further improve posttransplant outcomes [54, 55]. In this meta-analysis, the pooled results from three studies showed that an AFP level > 400 IU/mL at the time of transplantation was associated with a significant increase in the recurrence rate [12, 36, 38].

Additionally, this study illustrates that the presence of MVI increases the recurrence and mortality rate after transplantation. In addition to that, according to Lim et al. [16], HCC patients exceeding the MC without MVI could achieve comparable overall survival rates after surgical resection, relative to patients within Milan. In other words, to improve survival and decrease recurrence after transplantation, radiological tools are needed to predict the presence of MVI before liver transplantation [56, 57]. Moreover, macrovascular invasion of hepatic or portal veins has been documented in up to one-third of patients with hepatocellular carcinoma (HCC) [58]. According to AASLD Guidelines it is considered a contraindication to liver transplantation [59]. In our study, the presence of macrovascular invasion is associated with a dramatic increase in the recurrence rate and a significant decrease in survival. Thus it can be said that those patients could benefit from down staging [60].

Moreover, tumor grade of differentiation had a statistically significant effect on the long-term prognosis of HCC after LT. This is explained by Pawlik et al. that the grade was the most powerful predictor of occult vascular invasion [17]. Therefore, the role of percutaneous biopsy for grading prior to transplantation requires study as a way to improve outcomes.

To our knowledge, it is the first time for 2-, 4-, 6-, 10-year outcomes and predictors of recurrence after liver transplantation to be included in a meta-analysis. In addition to that, all studies designed to compare the outcome between LDLT and DDLT for HCC patients were included to increase the statistical power of the results.

However, we have to acknowledge some limitations in our study. First, all the studies included were cohort studies because no randomized controlled trials could be found. Second, the existence of significant heterogeneity in several outcomes could not be explained well enough by subgroup analysis. Third, included studies were conducted in different regions where policies and ethics about LT were different, and this might cause potential bias.

## Conclusion

This study is in consonance with the view that cancer biology (not the graft type) is the most important determinant of recurrence and survival after LT. However, LDLT provided much better survival benefits to HCC patients especially in regions that suffer from low deceased organ availability.

## Supplementary Information

Below is the link to the electronic supplementary material.

Supplementary file1 (PDF 280 kb)

## Abbreviations

AASLD: American association of study of liver disease
AFP: Alfa fetoprotein
DDLT: Deceased donor liver transplant
DFS: Disease free survival
HBV: Hepatitis B virus
HCC: Hepatocellular carcinoma
HCV: Hepatitis C virus
HDV: Hepatitis D virus
ITT-OS: Intention to treat overall survival
LDLT: Living donor liver transplant
LT: Liver transplant
MC: Milan criteria
N/A: Not applicable or not available
OS: Overall survival
VI: Vascular invasion
MVI: Microvascular invasion
