# Supplementary material for: Can living donor liver transplantation provide similar outcomes to deceased-donor liver transplantation for hepatocellular carcinoma? A systematic review and meta-analysis
Source: Hepatol Int. 2022 Dec 23;17(1):18–37. doi: 10.1007/s12072-022-10435-3 (PMC9894961; doi:10.1007/s12072-022-10435-3)

1-year OS

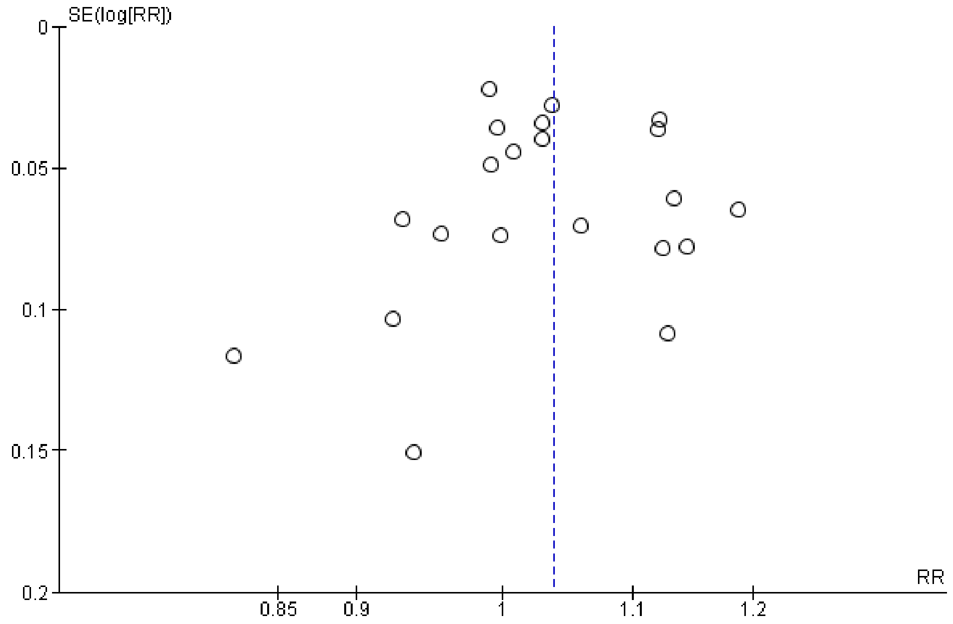

2-year OS

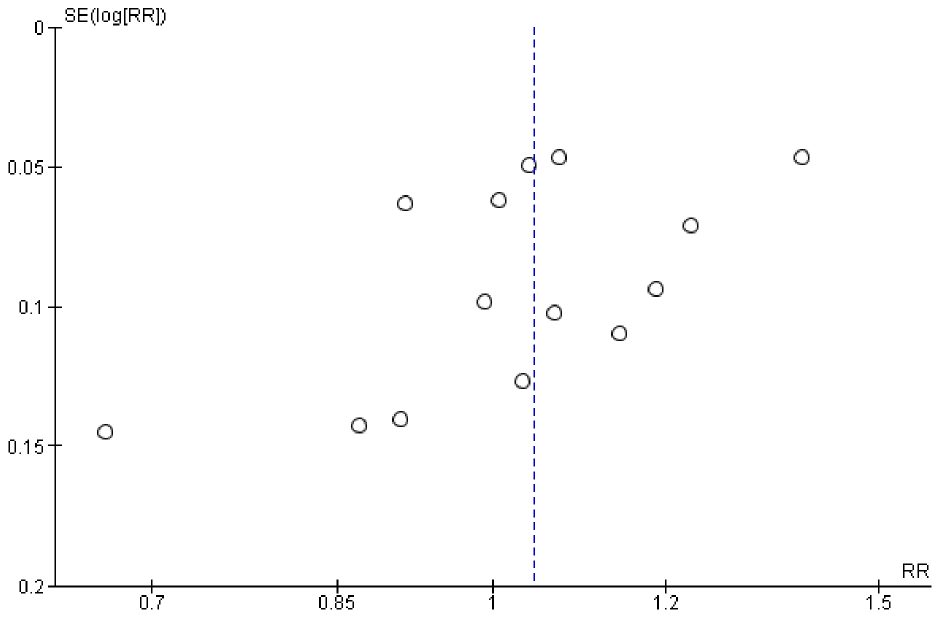

3-year OS

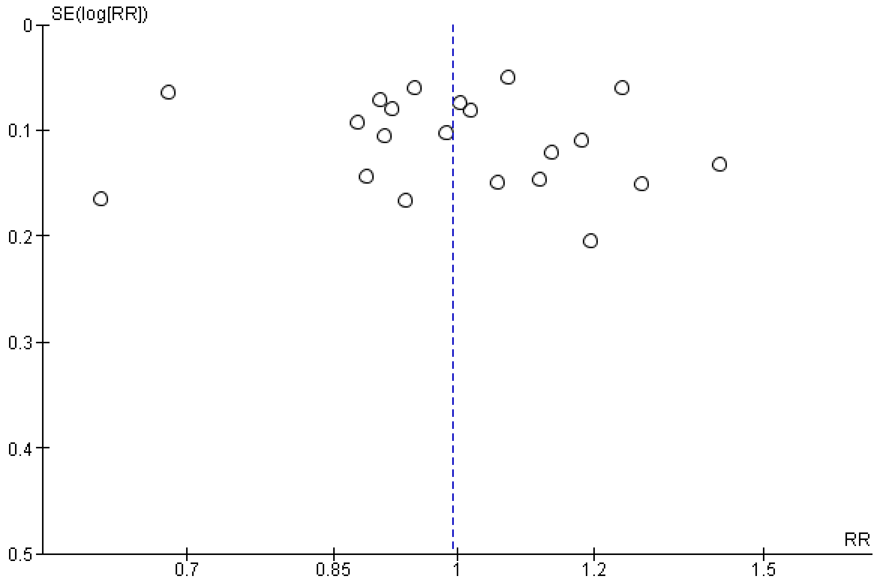

4-year OS

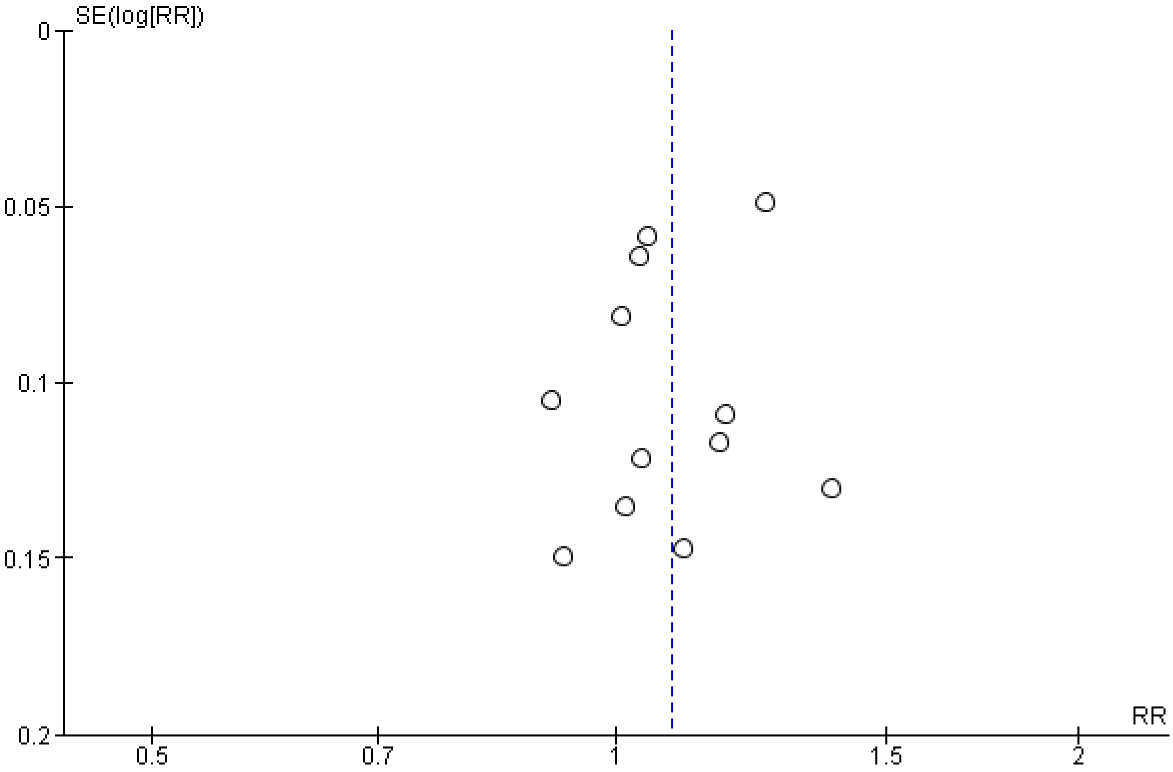

5-year OS

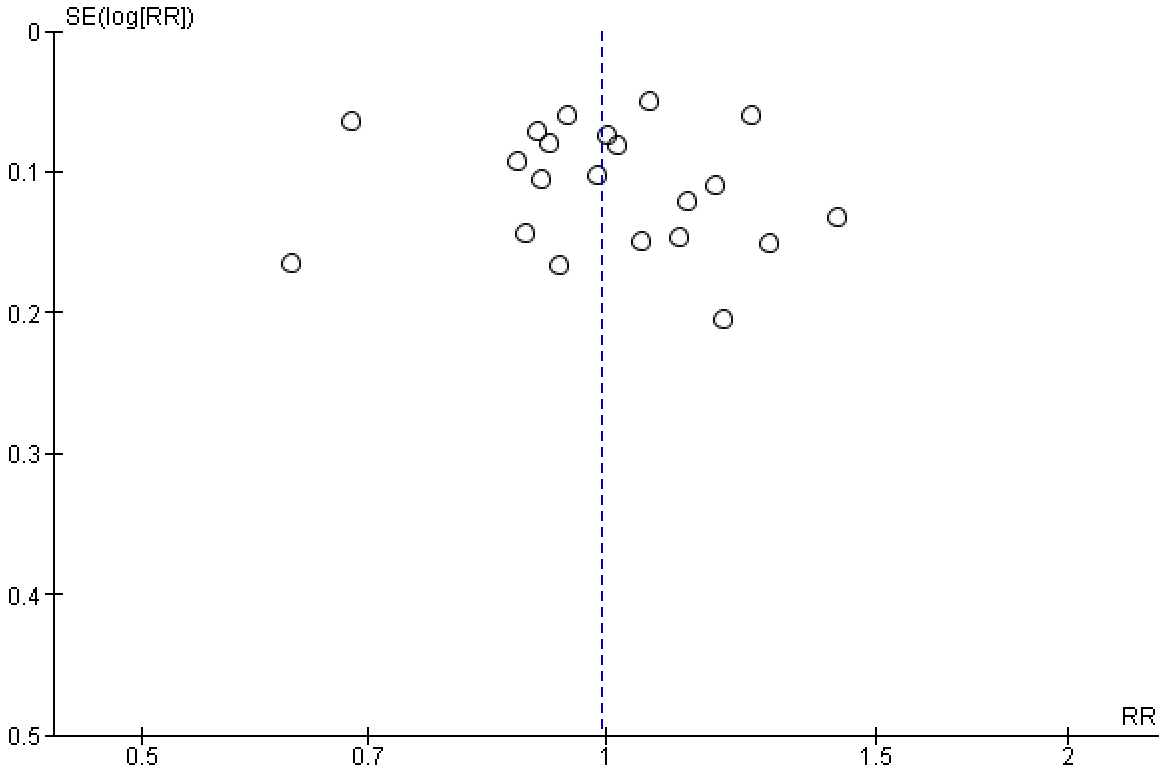
SE(log[RR])
0
0.1
0.2
0.3
0.4
0.5
RR
0.5
0.7
1
1.5
2

6-year OS

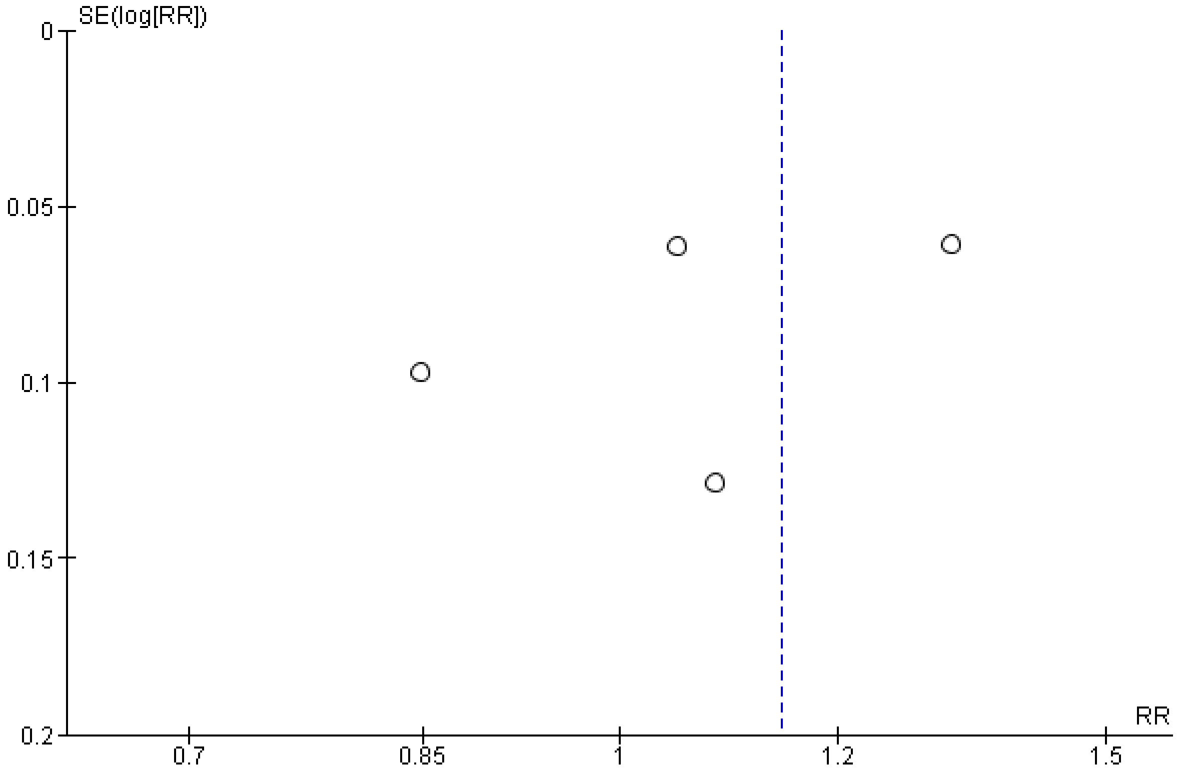
SE(log[RR])
0
0.05
0.1
0.15
0.2
RR
0.7
0.85
1
1.2
1.5

10-year OS

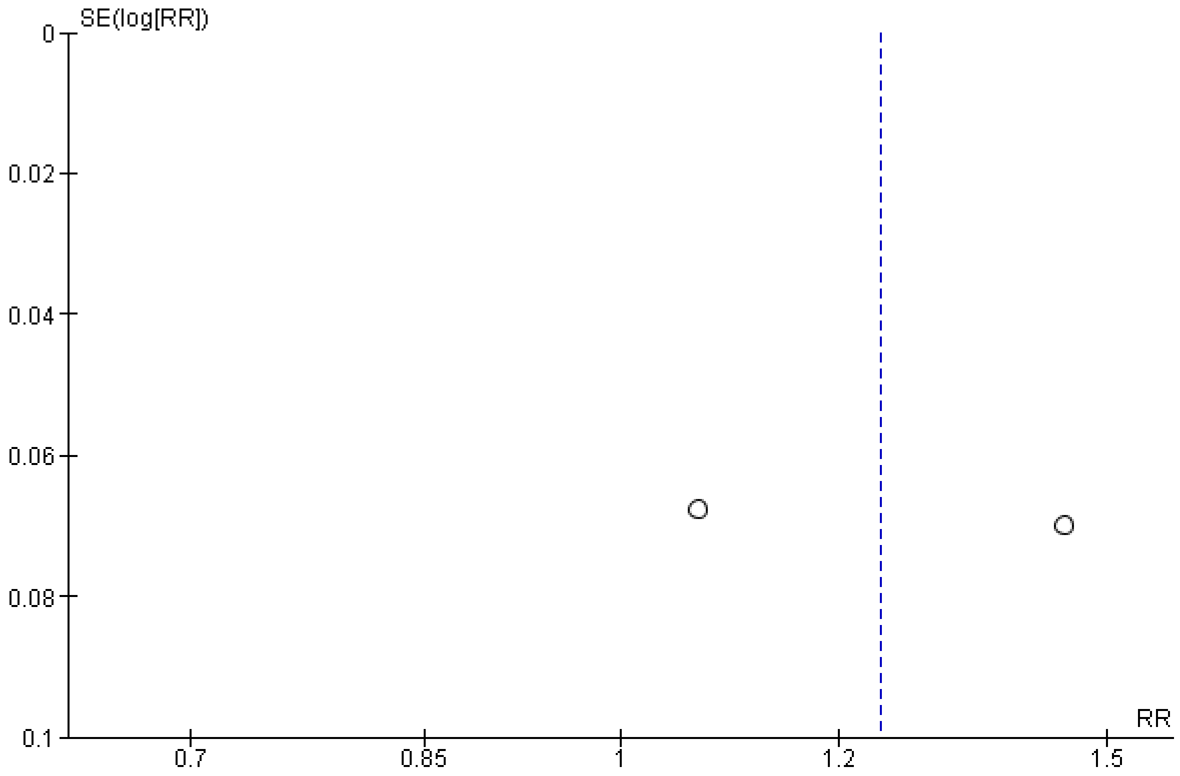

1-year DFS

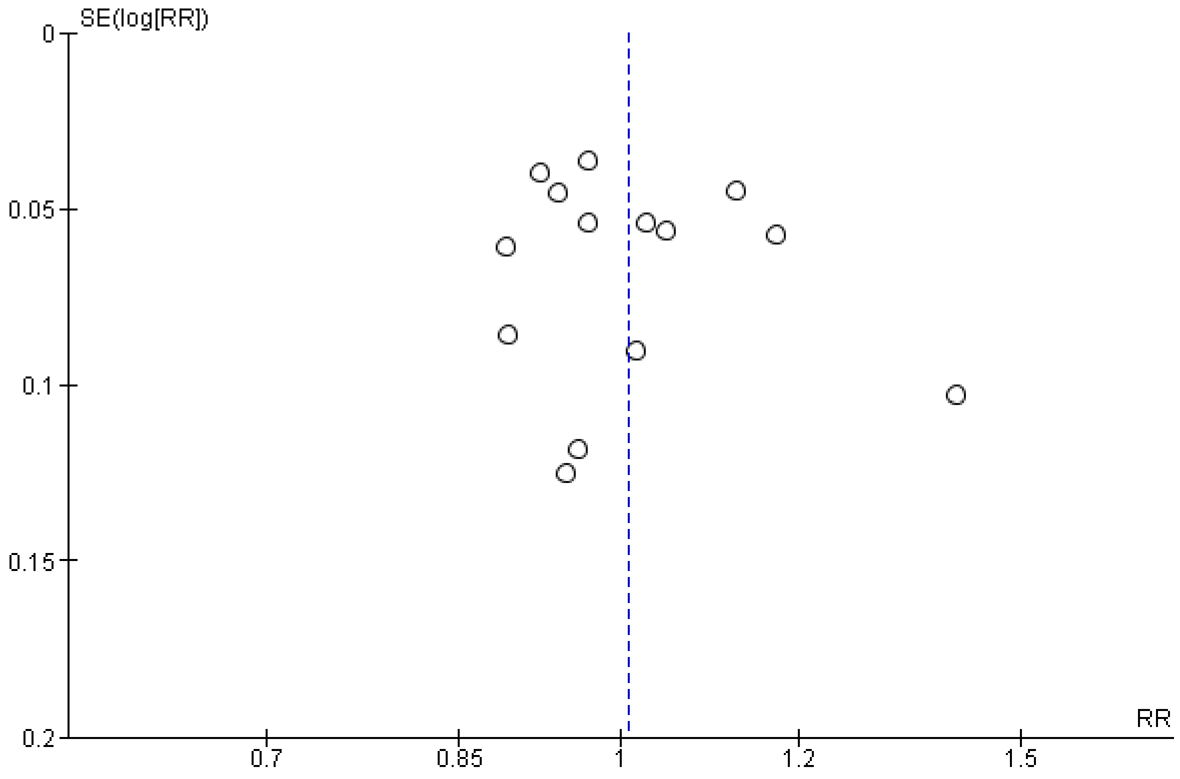

2-year DFS

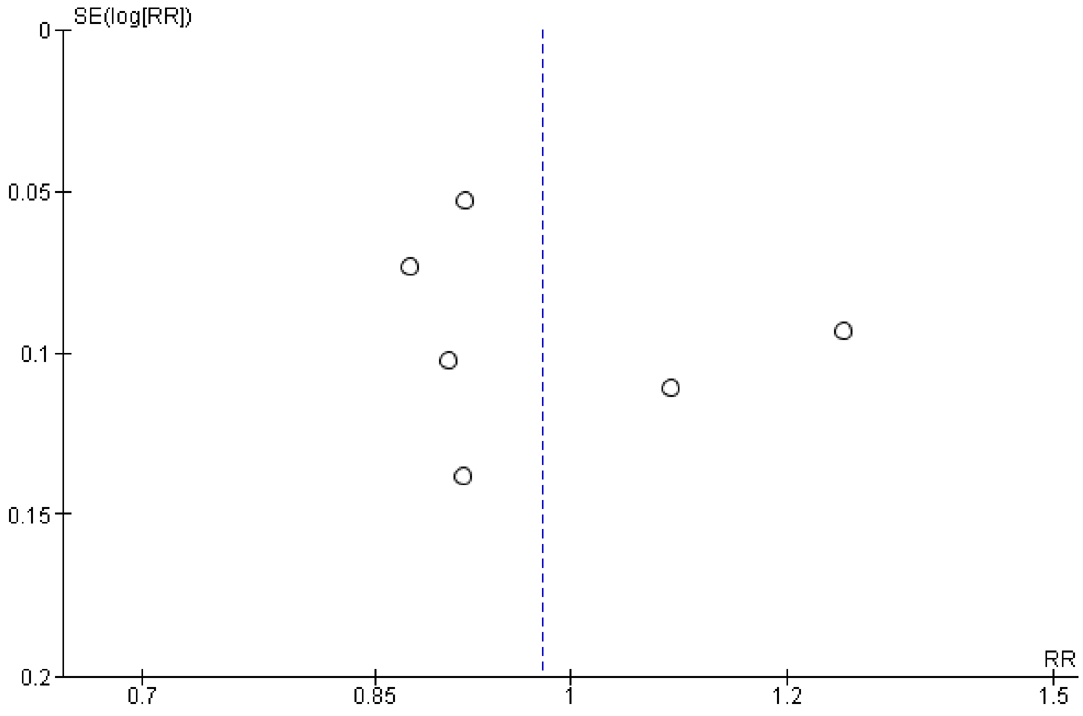

3-year DFS

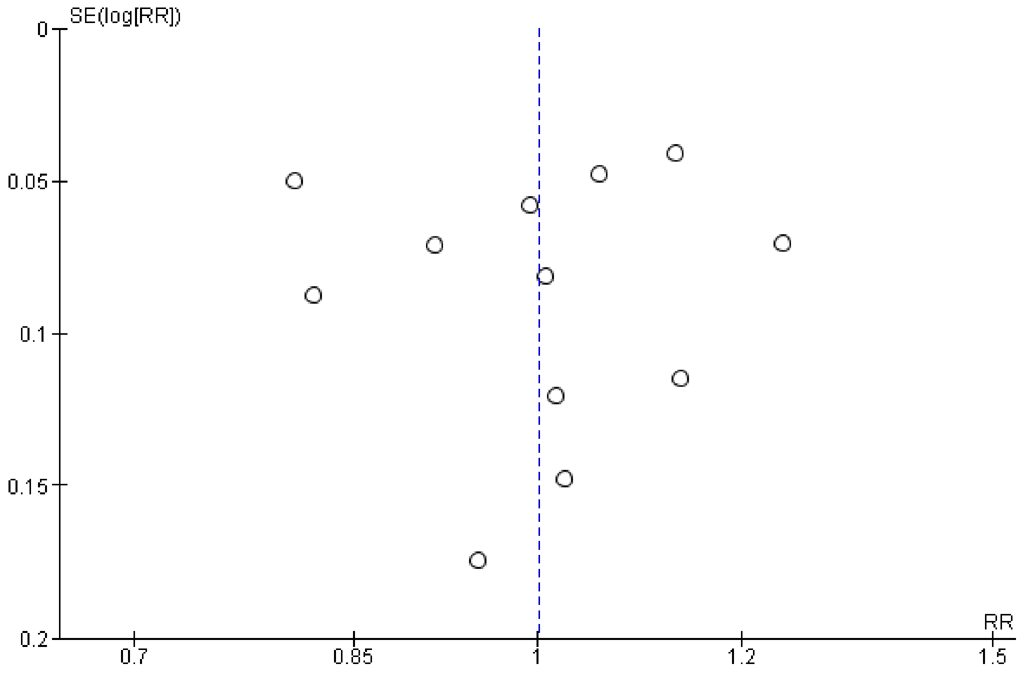

4-year DFS

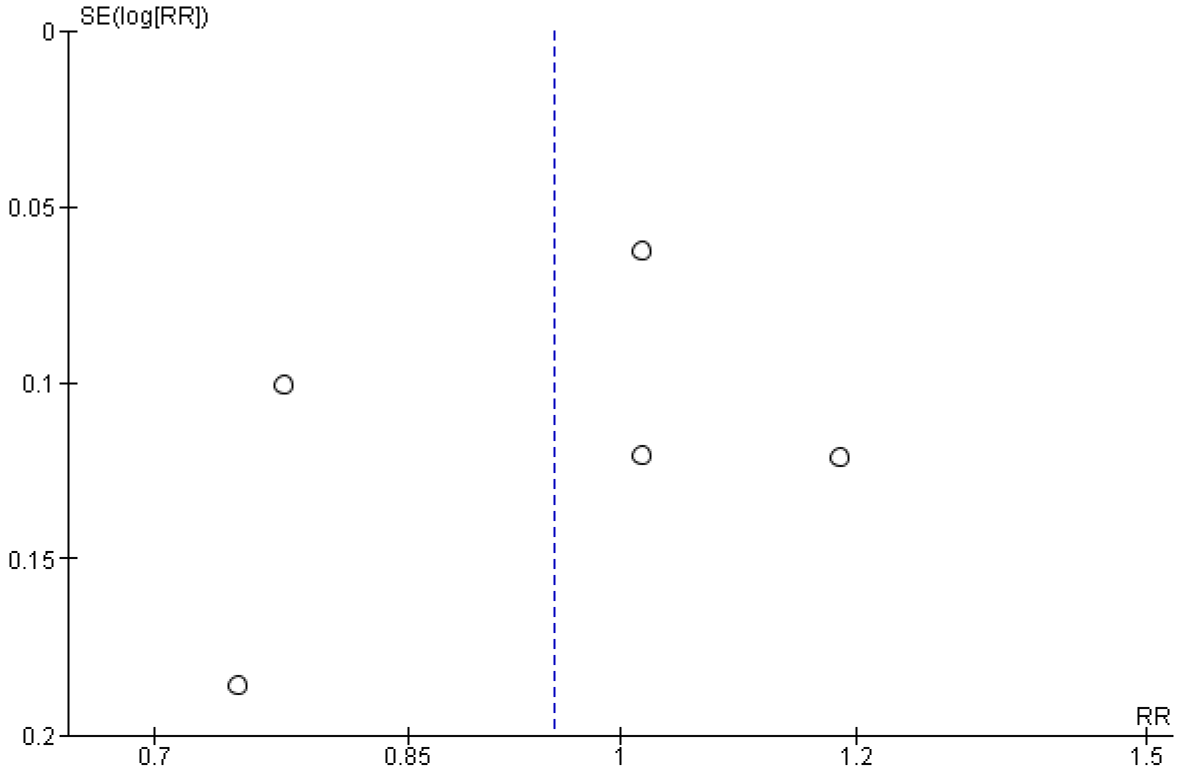

5-year DFS

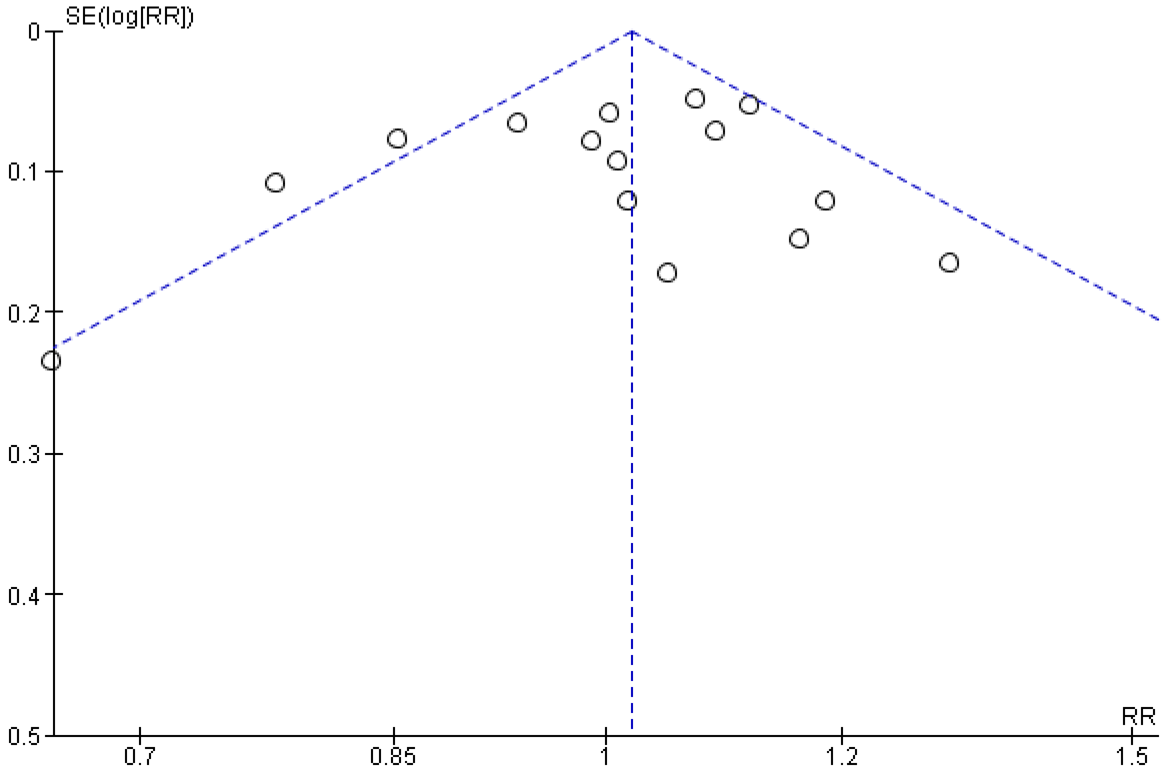

6-year DFS

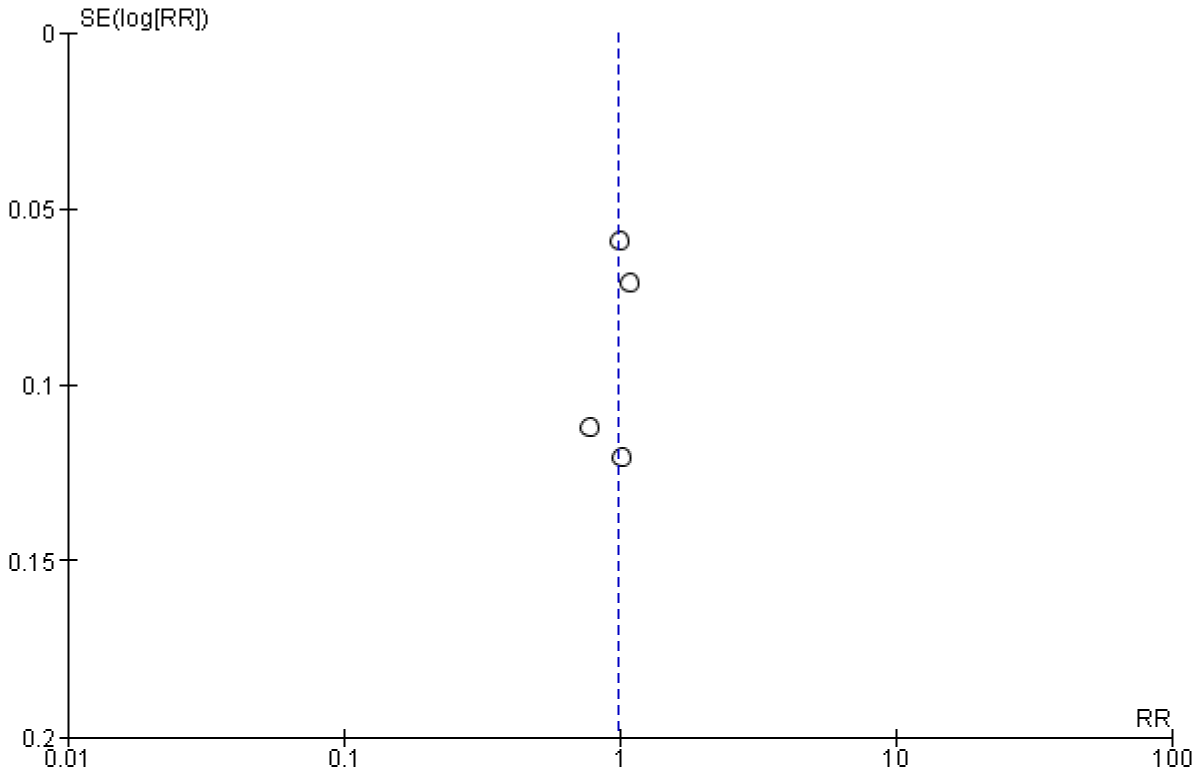

10-year DFS

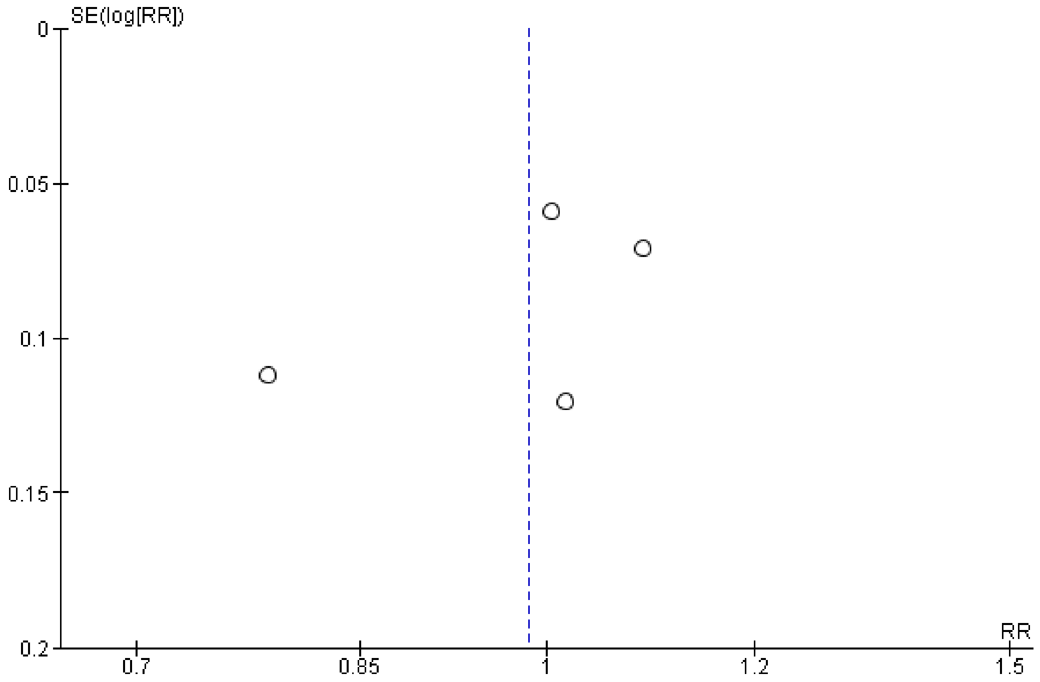

1-year ITT-OS

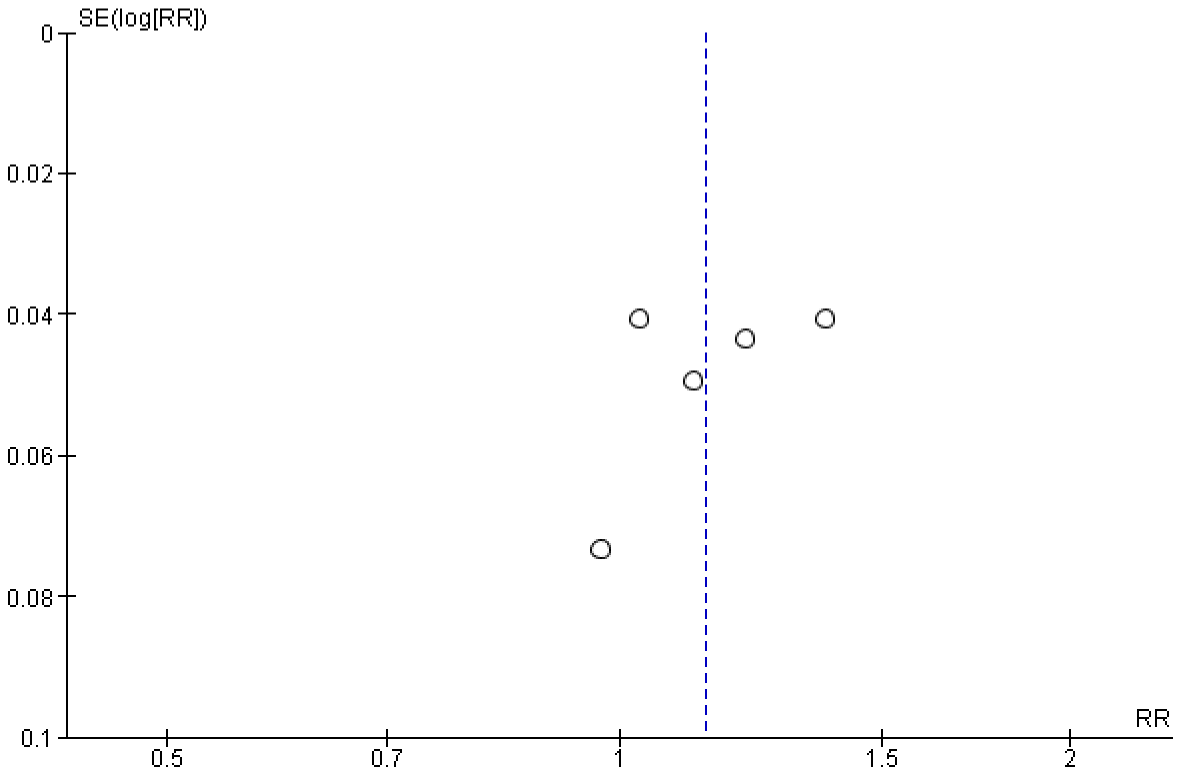

2-year ITT-OS

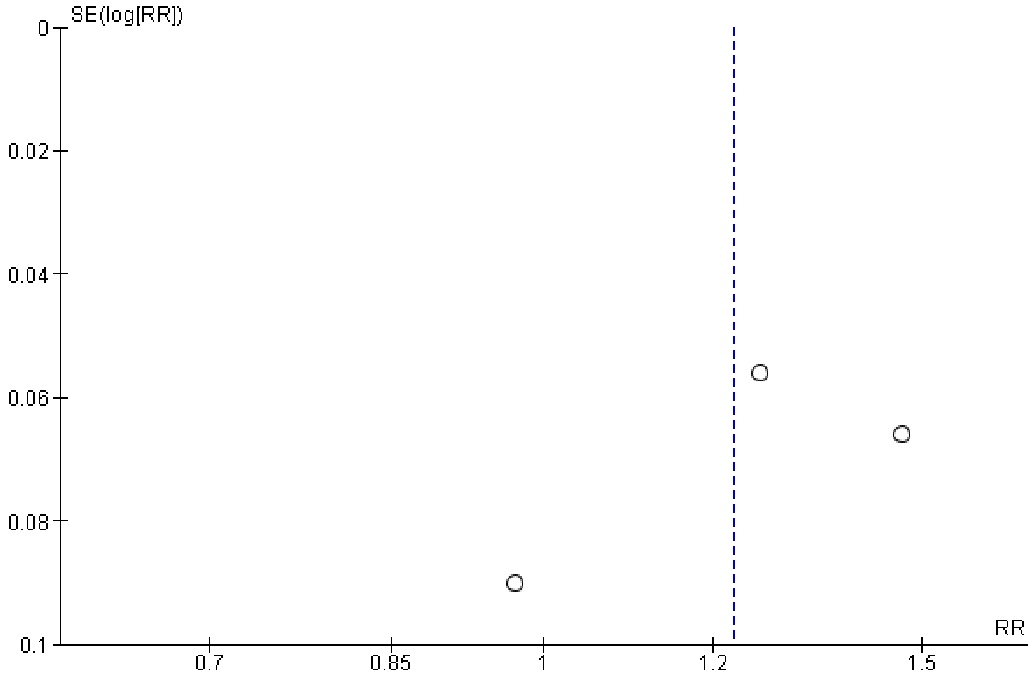

3-year ITT-OS

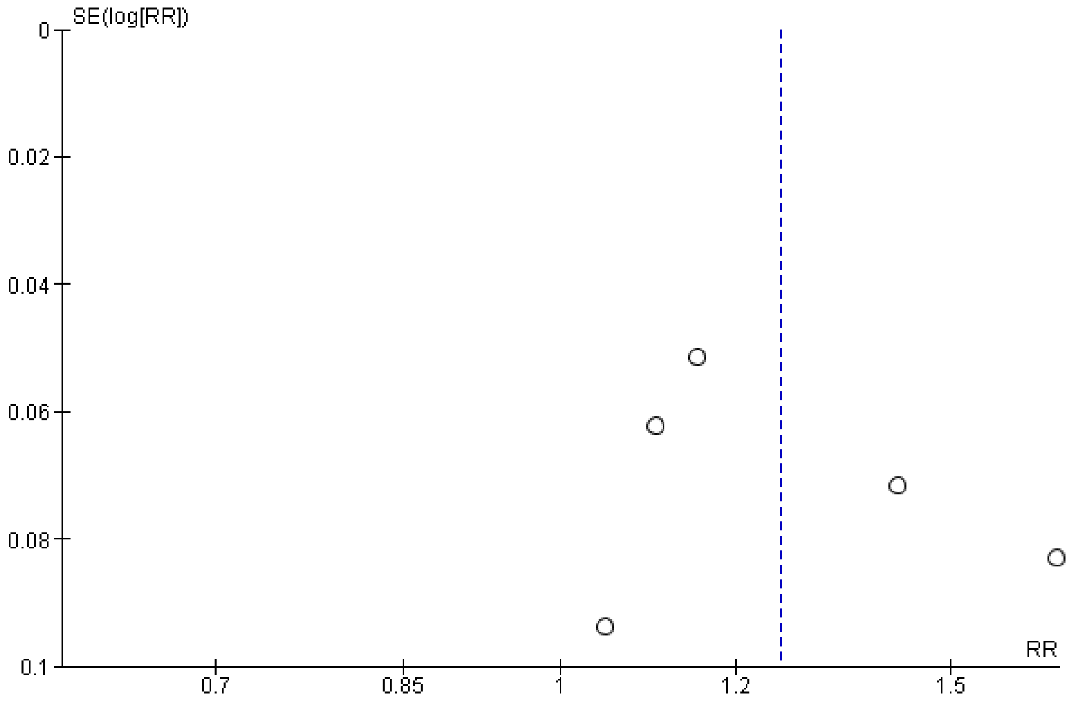

4-year ITT-OS

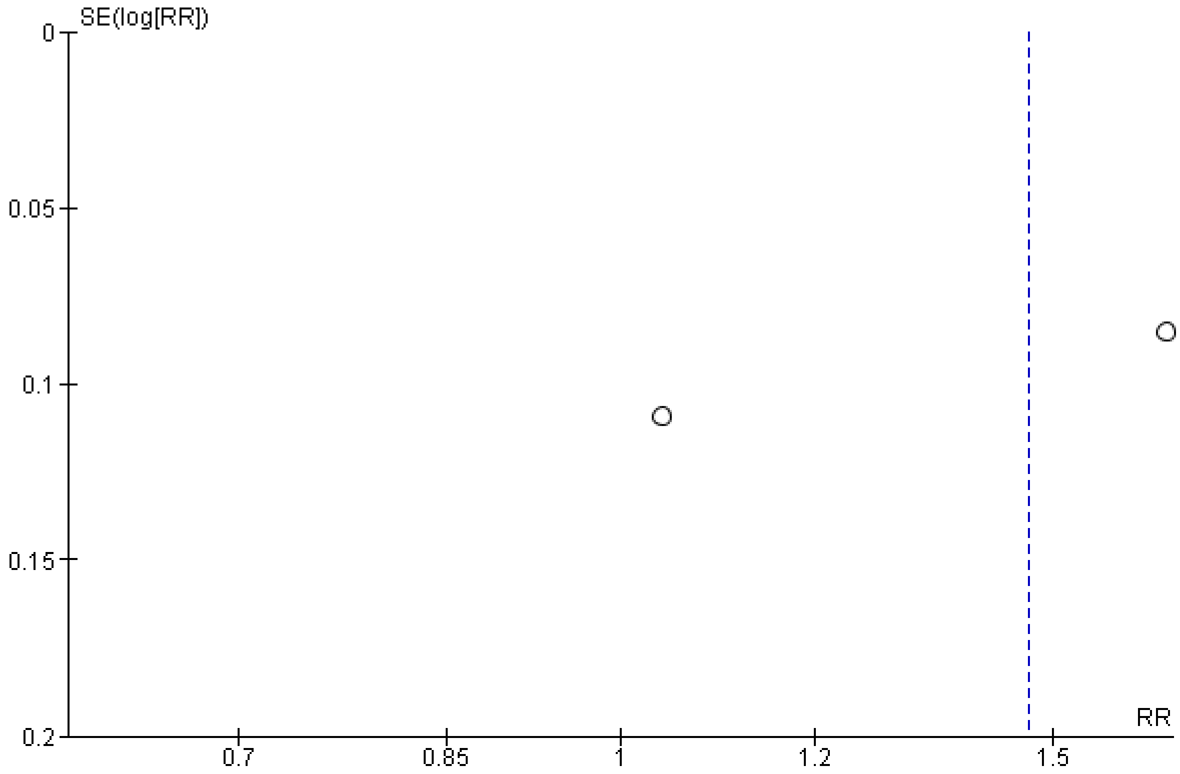

5-year ITT-OS

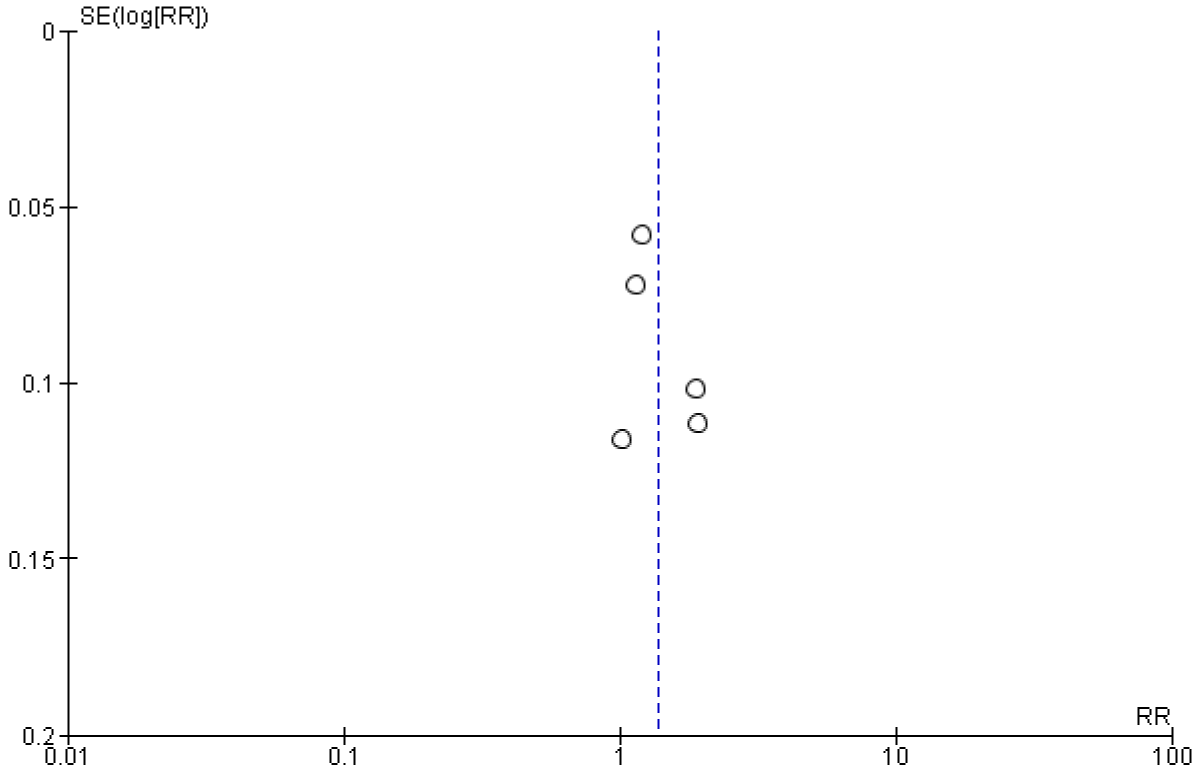

Recurrence for LDLT and DDLT recipients

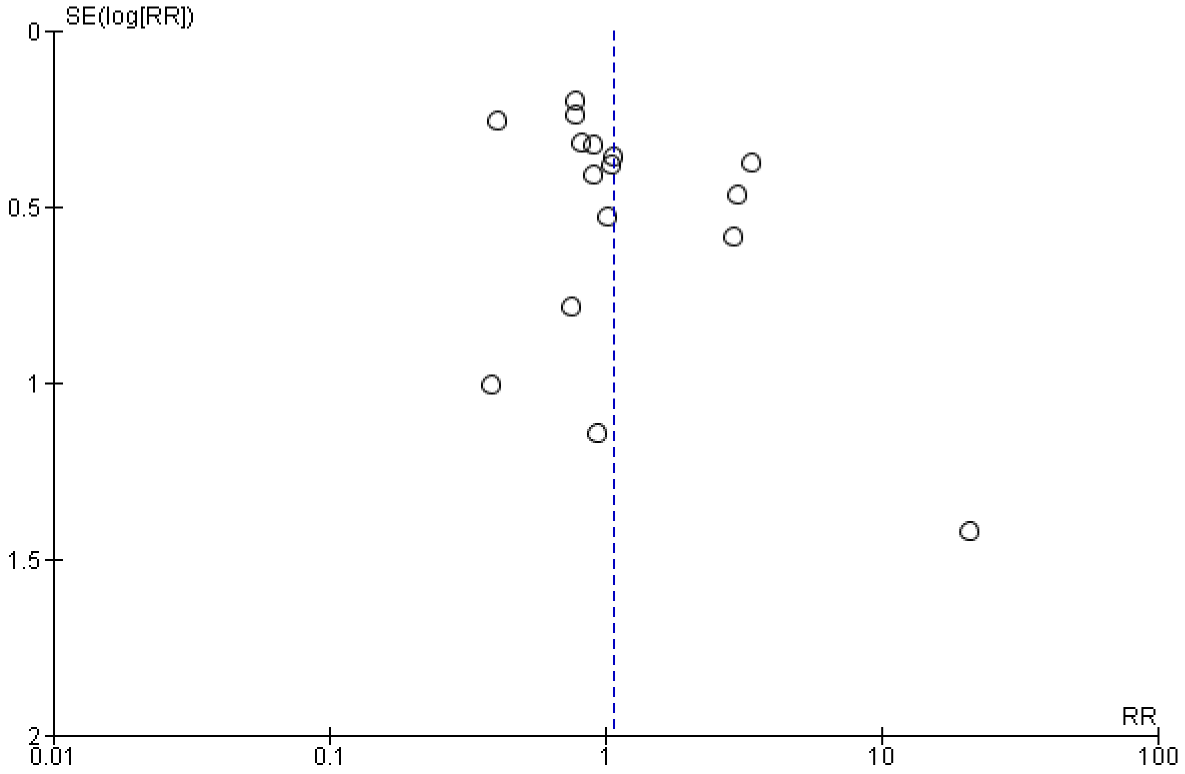

Supplement: Supplementary file 1 — Supplementary file1 (PDF 280 kb) [file 12072_2022_10435_MOESM1_ESM.pdf]
